# Non-muscle invasive bladder cancer tissues have increased base excision repair capacity

**DOI:** 10.1038/s41598-020-73370-z

**Published:** 2020-10-01

**Authors:** Berna Somuncu, Selcuk Keskin, Fatma Merve Antmen, Yesim Saglican, Aysegul Ekmekcioglu, Tugce Ertuzun, Mustafa Bilal Tuna, Can Obek, David M. Wilson, Umit Ince, Ali Riza Kural, Meltem Muftuoglu

**Affiliations:** 1 Department of Medical Biotechnology, Acibadem Mehmet Ali Aydinlar University, 34752 Atasehir Istanbul, Turkey; 2 Department of Urology, Acibadem Mehmet Ali Aydinlar University, 34752 Istanbul, Turkey; 3 Department of Pathology, Acibadem Mehmet Ali Aydinlar University, 34752 Istanbul, Turkey; 4 Department of Urology, Acibadem Maslak Hospital, Istanbul, Turkey; 5 Department of Urology, Acibadem Taksim Hospital, Istanbul, Turkey; 6 Biomedical Research Institute, Hasselt University, 3590 Diepenbeek, Belgium; 7 Department of Molecular Biology and Genetics, Acibadem Mehmet Ali Aydinlar University, 34752 Istanbul, Turkey

**Keywords:** Bladder cancer, DNA repair enzymes, Base excision repair, Bladder cancer

## Abstract

The molecular mechanisms underlying the development and progression of bladder cancer (BC) are complex and have not been fully elucidated. Alterations in base excision repair (BER) capacity, one of several DNA repair mechanisms assigned to preserving genome integrity, have been reported to influence cancer susceptibility, recurrence, and progression, as well as responses to chemotherapy and radiotherapy. We report herein that non-muscle invasive BC (NMIBC) tissues exhibit increased uracil incision, abasic endonuclease and gap-filling activities, as well as total BER capacity in comparison to normal bladder tissue from the same patient (*p* < 0.05). No significant difference was detected in 8-oxoG incision activity between cancer and normal tissues. NMIBC tissues have elevated protein levels of uracil DNA glycosylase, 8-oxoguanine DNA glycosylase, AP endonuclease 1 and DNA polymerase β protein. Moreover, the fold increase in total BER and the individual BER enzyme activities were greater in high-grade tissues than in low-grade NMIBC tissues. These findings suggest that enhanced BER activity may play a role in the etiology of NMIBC and that BER proteins could serve as biomarkers in disease prognosis, progression or response to genotoxic therapeutics, such as Bacillus Calmette–Guérin.

## Introduction

Bladder cancer (BC) is the ninth most common cancer worldwide^1^. Non-muscle invasive BC (NMIBC) is responsible for approximately 75–85% of newly diagnosed BCs and includes tumors with pathological stages Ta (noninvasive papillary carcinoma), T1 (invasive into the subepithelial connective tissue), and carcinoma in situ (CIS, flat tumor). The recurrence rate of NMIBC is 30–80%, and 10–20% of the recurrent cases progress to muscle-invasive BC (MIBC) (stages ≥ T2) within 2–5 years. Several studies have reported that grade is a better prognostic indicator for NMIBC progression and mortality, because patients with high-grade tumors progress with similar frequency regardless of whether they were invasive (T1 stage) or noninvasive (Ta stage)^2–4^. The molecular mechanisms underlying the development and progression of BC are complex and have not been completely defined. Lifestyle choices and environmental agents, such as smoking, exposure to certain industrial aromatic amines or polycyclic hydrocarbons, and consumption of water that is contaminated with arsenic, are important risk factors for NMIBC^3^. Because only a subset of the individuals exposed to these factors develop NMIBC, there likely exists different hereditary and/or predisposing genetic factors for the disease.

Notably, many of the environmental risk factors for NMIBC increase the production of reactive oxygen species (ROS), which generate oxidative damage to cellular macromolecules, including DNA. Base excision repair (BER) is a major protective pathway against the adverse effects of oxidative, alkylative and hydrolytic DNA damage, generated by both endogenous (such as ROS) and exogenous sources^5,6^. Not surprisingly, a defect in BER, particularly in the face of genotoxic stress, can lead to the accumulation of DNA damage and genomic instability, and has been linked to genetic disorders exhibiting cancer proneness^7–9^. More broadly speaking, DNA repair rate/capacity, which is different among individuals and tumors, is an important phenotype that contributes to the inter-individual variability in cancer development and therapeutic response^10–13^.

BER is initiated by several lesion-specific DNA glycosylases that recognize and remove inappropriate or damaged bases. For example, uracil DNA glycosylase (UDG) removes uracil from the genome, and 8-oxoguanine DNA glycosylase (OGG1) excises 8-hydroxy-7,8-dihydroguanine (8-oxoG) from DNA. After the modified base is removed, the resulting apurinic/apyrimidinic (AP) site is cleaved primarily by the major AP endonuclease, APE1. Subsequently, BER proceeds either through single nucleotide gap-filling by DNA polymerase *β* (POLβ) (short-patch) or long-patch repair synthesis (2–13 nt) that commonly involves the replicative DNA polymerases δ/ε. Following termini clean-up by either POLβ or the 5′-flap structure-specific endonuclease 1 (FEN1), the remaining nick is sealed by a DNA ligase 3 (LIG3) and X-ray repair cross-complementing protein 1 (XRCC1) complex or LIG1^6^.

Several BER genes, including *APE1*, *POLβ* and *XRCC1*, are overexpressed in multiple cancer types, such as prostate, cervical, non-small cell lung, ovarian, glioma, triple-negative breast and BCs^14–33^, suggesting that alterations in BER gene expression can influence disease susceptibility, initiation, and progression. High expression levels of the APE1/REF1 protein in serum, urine, and tissue samples of NMIBC and MIBC patients that have not received chemotherapy or radiotherapy are indeed associated with the progression of BC^16,22,23^. Additional studies on the role of BER in NMIBC and MIBC have focused on variant profiles^9,34–41^ of BER genes, including *UNG*, *OGG1*, *APE1*, single-strand-selective monofunctional uracil-DNA glycosylase 1 (*SMUG1*), thymine-DNA glycosylase (*TDG*), MutY DNA glycosylase (*MUTYH*), and nei like DNA glycosylase 1 (*NEIL1*), *NEIL2*, and *XRCC1*. The work of Wei et al. has found that the *UNG* rs3890995 variant is significantly associated with NMIBC progression, while the *NEIL2* rs804256 variant is associated with recurrence in the Bacillus Calmette–Guérin (BCG)-treated NMIBC group^34^. In a separate study, it was reported that the SNP most significantly associated with BC risk is *SMUG1* rs2029167^36^. The *APE1* Asp148Glu variant has also been associated with increased^35^ and decreased^39^ risks of BC. Studies with large sample sizes are now needed to verify the above observations. To further define the role of BER in NMIBC, we measured total BER and individual BER protein activities and levels in whole lysates prepared from NMIBC tissue and normal tissue from the same individual.

## Results

Total BER activity, uracil incision, 8-oxoG incision, AP endonuclease and gap-filling activities were measured in seventeen NMIBC tissues in comparison to normal bladder tissue from the same patient. Before performing each BER activity assay, we determined the optimal tissue extract protein concentration using increasing amounts of whole tissue extracts of NMIBC and the corresponding normal tissues as indicated in Supplementary Fig. S1a–S5a. As seen, each activity increased in a protein concentration-dependent manner, and at each concentration examined, the activity was higher in NMIBC tissue than in the corresponding normal tissue (Supplementary Fig. S1a–S5a). In addition to tissue extracts, we also measured BER activities of T24 cell extracts (Supplementary Fig. S1b–S3b; S4c and S5b).

### Increased total BER activity in NMIBC tissues

**Figure 1:**
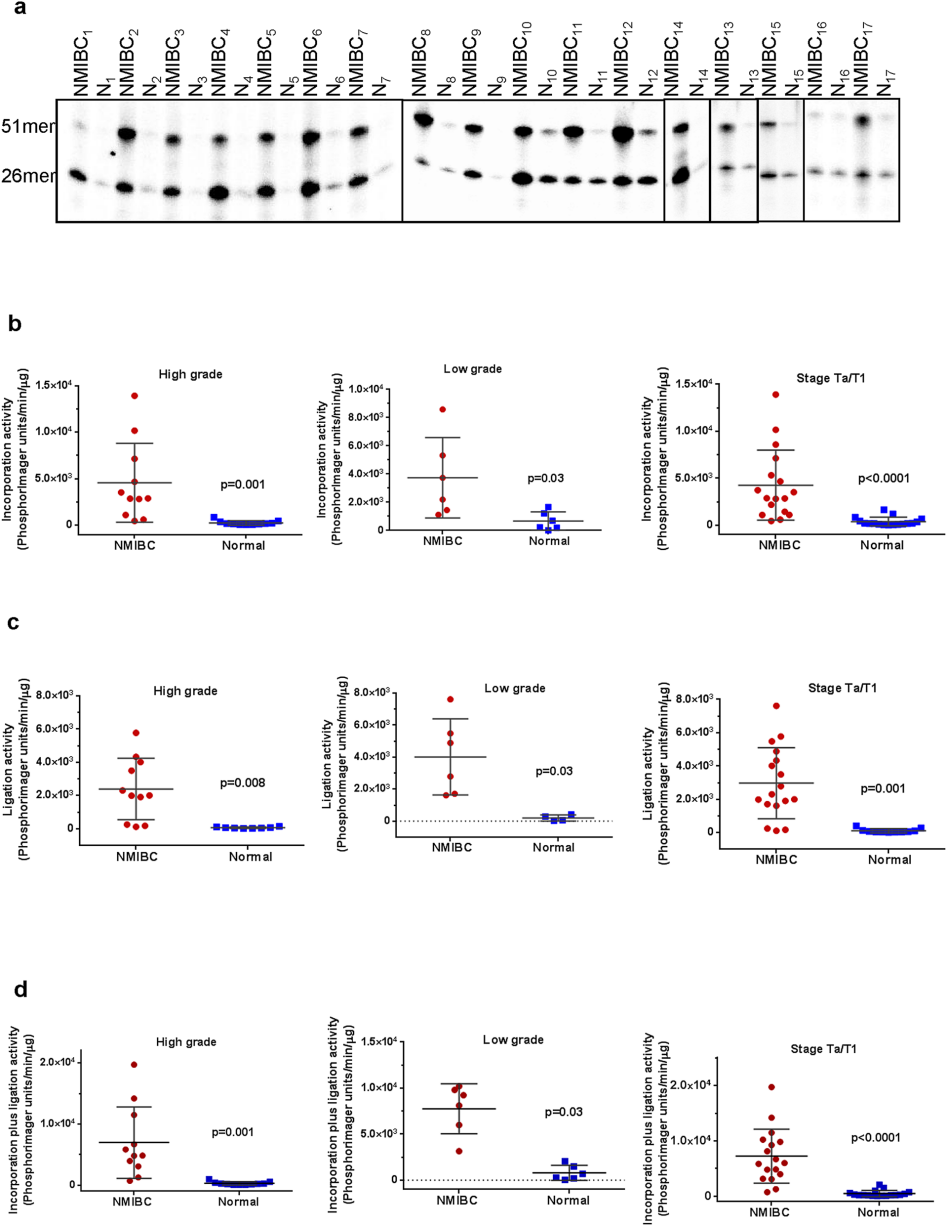
Uracil-initiated total BER activity in NMIBC and their corresponding normal tissues. (**a**) Representative gel for uracil-initiated total BER activity showing products of ^32^P-dCTP incorporation (26mer) and ligation (51mer). The lower band represents 1-nt incorporation product (26mer) and the upper band represents the 51mer ligated product*.* N, the corresponding normal tissue. Quantitation of incorporation (**b**), ligation (**c**) and incorporation plus ligation (**d**) activities from low-grade, high-grade, and Ta/T1 stage NMIBC and their corresponding normal tissues. The incorporation, ligation and incorporation plus ligation activities are presented as PhosphorImager units per min per μg protein. Low-grade sample numbers are 2, 4, 9–12; high-grade sample numbers are 1, 3, 5–8, 13–17. The full-length gels are presented in Supplementary Fig. 1. Gels were visualized by Typhoon FLA 9500 PhosphorImager and analyzed using the ImageQuant software TL v8.1 (https://www.cytivalifesciences.com/en/us/shop/protein-analysis/molecular-imaging-for-proteins/imaging-software/imagequant-tl-8-1-p-00110).

**Figure 2:**
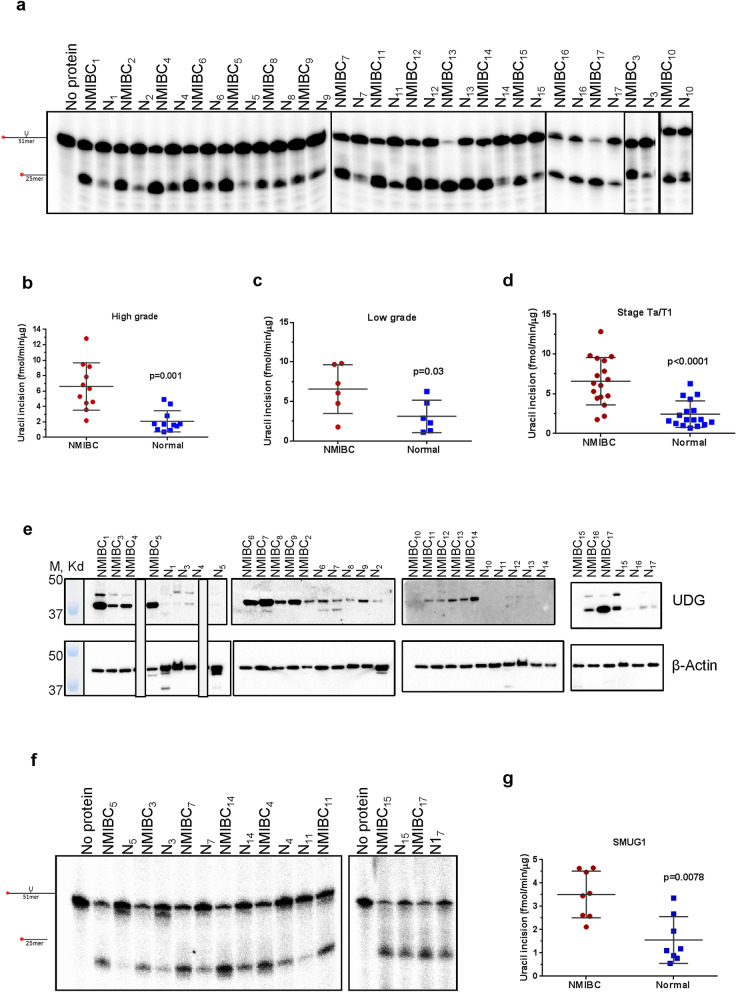
Uracil incision activities and UDG protein levels in NMIBC and their corresponding normal tissues. (**a**) Representative gel for uracil incision. Incision products are 25mer derived from cleaved ^32^P-labeled 51mer DNA substrate containing uracil at position 26. N, the corresponding normal tissue. Quantitation of uracil incision from high-grade (**b**), low-grade (**c**) and Ta/T1 stages (**d**) of all NMIBC and their corresponding normal tissues (fmol/μg/min). (**e**) Western blot analysis of UDG and β-actin in NMIBC and their corresponding normal tissues. Low-grade sample numbers are 2, 4, 9–12; high-grade sample numbers are 1, 3, 5–8, 13–17. (**f**) Representative gel for single-stranded uracil incision. (**g**) Quantitation of single-stranded uracil incision. The full-length gels/blots are presented in Supplementary Fig. 2. Radioactive gels were visualized by Typhoon FLA 9500 PhosphorImager and analyzed using the ImageQuant software TL v8.1 (https://www.cytivalifesciences.com/en/us/shop/protein-analysis/molecular-imaging-for-proteins/imaging-software/imagequant-tl-8-1-p-00110).

To compare the BER capacity of NMBIC tissue with normal bladder tissue from the same individual (of seventeen patients, Table 1), whole tissue lysates were prepared and uracil-initiated total BER activity was measured. In this assay, we determined the efficiency of incorporation of ^32^P-dCMP in place of a substrate uracil (at position 26, U/G base pair) within a 51mer duplex (Fig. 1a, 26mer band) and subsequent ligation of the nicked intermediate (Fig. 1a, 51mer band). A representative gel for uracil-initiated total BER activity using whole tissue extracts prepared from NMIBC and the corresponding normal tissue is shown in Fig. 1a; the lower band represents the 1nt-^32^P-dCMP insertion product (26mer) and the upper band represents the 51mer final ligated repair product. Importantly, β-actin protein levels were similar in the NMIBC and corresponding normal tissue protein extracts used in the activity assays (see for example Fig. 2e), indicating that any differences measured would reflect true differences in the repair activity interrogated. Moreover, when whole tissue extracts were incubated with an undamaged control substrate (Table 2), no product was observed (data not shown), demonstrating the specificity of the assays selected. Quantification of the BER capacity experiments found that the incorporation (Fig. 1b), the ligation (Fig. 1c), and the incorporation plus ligation activities (Fig. 1d) were significantly (*p* < 0.05) higher in both low-grade and high-grade NMIBC tissues as compared to their corresponding normal tissues. All NMIBC tissues classified as Ta/T1 stage (n = 17) had enhanced BER activity compared to the normal tissue sample (*p* < 0.0001) (Fig. 1b,d). Direct comparison of each NMIBC tissue separately to the normal tissue of the same patient is shown in Supplementary Table S1 online. Consistent with the collective results, we see up-regulation in total BER capacity in each NMIBC tissue compared to its corresponding normal material (Supplementary Table S1 online). Notably, high-grade NMIBC tissues have higher total BER activity compared to low-grade NMIBC tissues (Fig. 1, Supplementary Table S1 online).

**Table 1:** Characteristics of the NMIBC patients. Since control tissues are from the same person (the corresponding normal tissue), number, age and gender properties are the same as for NMIBC tissue*.

Characteristics	
Number of patients	17
Gender (male/female) (n/n)	14/3
Mean age (years)	68.65 ± 15.02
Age range (years)	37–92
Diagnosis (n)	NMIBC, transitional cell carcinoma (17)
Grade (n)	Low-grade (6); High-grade (11)
Stage (n)	Ta (14); T1 (3)
Primer/recurrence	primer
Treatment	None
Control (n)	Normal bladder tissue* (17)

**Table 2:** Oligodeoxynucleotide sequences used in this study.

Oligodeoxynucleotides
**U = Uracil** 5′-GCTTAGCTTGGAATCGTATCATGTAUACTCGTGTGCCGTGTAGACCGTGCC-3′ 3′-CGAATCGAACCTTAGCATAGTACATGTGAGCACACGGCACATCTGGCACGG-5′
^**OH**^**G = 8-oxoG** 5′-GCTTAGCTTGGAATCGTATCATGTA^OH^GACTCGTGTGCCGTGTAGACCGTGCC-3′ 3′-CGAATCGAACCTTAGCATAGTACAT GTGAGCACACGGCACATCTGGCACGG-5′
^**OH**^**G = 8-oxoG** 5′-GCTTAGCTTGGAATCGTATCATGTA^OH^GACTCGTGTGCCGTGTAGACCGTGCC-3′ 3′-CGAATCGAACCTTAGCATAGTACAT CTGAGCACACGGCACATCTGGCACGG-5′
**X = Tetrahydrofuran** 5′-GCTTAGCTTGGAATCGTATCATGTAXACTCGTGTGCCGTGTAGACCGTGCC-3′ 3′-CGAATCGAACCTTAGCATAGTACATGTGAGCACACGGCACATCTGGCACGG-5′
**1nt-gap** 5′-GCTTAGCTTGGAATCGTATCATGTAACTCGTGTGCCGTGTAGACCGTGCC-3′ 3′-CGAATCGAACCTTAGCATAGTACATGTGAGCACACGGCACATCTGGCACGG-5′
**Control** 5′-GCTTAGCTTGGAATCGTATCATGTACACTCGTGTGCCGTGTAGACCGTGCC-3′ 3′-CGAATCGAACCTTAGCATAGTACATGTGAGCACACGGCACATCTGGCACGG-5′

### NMIBC tissues have increased uracil incision activity, but not 8-oxoG incision activity

**Figure 3:**
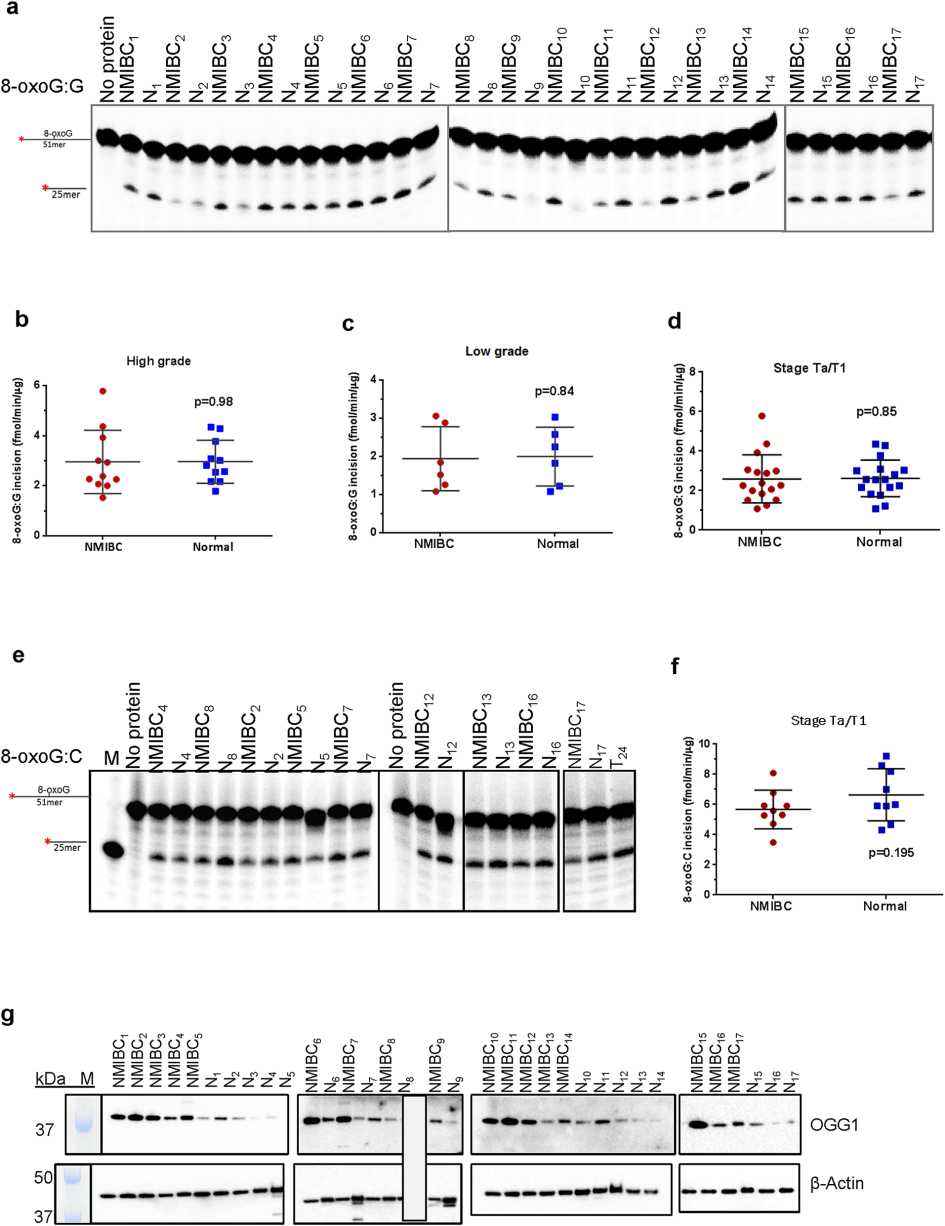
8-oxoG incision activity and OGG1 protein levels in NMIBC and their corresponding normal tissues. (**a**) Representative gel for 8-oxoG incision. Incision products are 25mer derived from cleaved ^32^P-labeled 51mer DNA substrate containing uracil 8-oxoG at position 26. N, the corresponding normal tissue. Quantitation of 8-oxoG incision from high-grade (**b**), low-grade (**c**) and Ta/T1 stages (**d**) of all NMIBC and their corresponding normal tissues (fmol/μg/min). (**e**) Representative gel for 8-oxoG:C incision. (**f**) Quantitation of 8-oxoG:C incision. (**g**) Western blot analysis of OGG1 and β-actin in NMIBC and their corresponding normal tissues. Low-grade sample numbers are 2, 4, 9–12; high-grade sample numbers are1, 3, 5–8, 13–17. The full-length gels/blots are presented in Supplementary Fig. 3. Radioactive gels were visualized by Typhoon FLA 9500 PhosphorImager and analyzed using the ImageQuant software TL v8.1 (https://www.cytivalifesciences.com/en/us/shop/protein-analysis/molecular-imaging-for-proteins/imaging-software/imagequant-tl-8-1-p-00110).

In follow-up to the uracil-initiated BER assay performed above, we next examined exclusively uracil incision activity in NMIBC and corresponding normal tissue extracts. In addition, 8-oxoG incision activity, predominantly performed by the DNA glycosylase, OGG1, was examined. Uracil and 8-oxoG repair activities were measured as the incision of double-stranded oligodeoxynucleotide substrates containing either a single uracil (U:G) or 8-oxoG lesion (8-oxoG:G and 8-oxoG:C) (Table 2). Uracil incision activity was also measured using a single-stranded oligodeoxynucleotide substrate containing uracil (ssU). An undamaged duplex oligodeoxynucleotide substrate (Table 2) was used as a negative control in both experiments, and no product was observed in either case (Supplementary Fig. S2b and S3b). The activities of U:G incision and 8-oxoG incision of the tissue extracts were effectively neutralized by UDG and OGG1 antibodies, respectively (Supplementary Fig. S2c and S3d). Co-incubation of tissue extracts with ugi completely inhibited the UDG activity (Supplementary Fig. S2d), but not the 8-oxoG incision activity (Supplementary Fig. S3c). The recombinant UDG and OGG1 proteins incised uracil and 8-oxoG in substrates completely (100% incision), respectively (Supplementary Fig. S2d and S3e). Thus, the collective data indicate that the observed uracil and 8-oxoG incision activities are attributable to UDG and OGG1, respectively. Representative gels for uracil and 8-oxoG incision in whole tissue extracts are shown in Fig. 2a and Fig. 3a, respectively.

Uracil incision activity (U:G substrate, mainly conducted by UDG) was found to be significantly higher in all high-grade (Fig. 2b, *p* = 0.001) and all low-grade (Fig. 2c, *p* = 0.03) NMIBC tissue extracts compared with the corresponding normal tissue. All NMIBC tissues at the Ta/T1 stage exhibited a significant increase in UDG activity (Fig. 2d, *p* < 0.0001). Comparisons of uracil incision between each NMIBC and its corresponding normal tissue are shown in Supplementary Table S1 online. Collectively, uracil incision activity was 4.02 ± 0.69 fold higher in high-grade and 2.65 ± 0.62 fold higher in low-grade NMIBC tissue (Fig. 2b,c, Supplementary Table S1 online). UDG protein levels of whole tissue extracts were at least 3.56 fold higher in NMIBC tissues than their corresponding normal tissues (Fig. 2e, Supplementary Table S2 online). We note that the UDG protein level of two normal tissues (N10 and N11) was found too low to detect with Western blot analysis (Fig. 2e, Supplementary Table S2 online).

To further address specificity of uracil incision activity, we performed assays using ssU substrates with eight NMIBC and corresponding normal tissue extracts, due to the limited amount of tissue extract available in all samples. Similar to what was seen in the U:G experiments, ssU incision activity was significantly higher in the eight NMIBC tissues (2.87 ± 0.71 fold) compared with the corresponding normal tissues (Fig. 2f,g and Supplemental Fig. S2f,g, *p* = 0.0078). Although SMUG1 is the major DNA glycosylase for ssU^42^, we could not detect the protein in these extracts by western blot analysis, suggesting that UDG is the main enzyme responsible for the uracil excision activity. There was a significant Pearson correlation between U:G incision activity and UDG protein level in NMIBC (r = 0.51, *p* = 0.04) but not in normal tissue (*p* = 0.18) (Supplementary Table S3 online).

Collectively, we found no significant difference in 8-oxoG incision (mainly performed by OGG1) activity in both high-grade (Fig. 3b, *p* = 0.98) and low-grade (Fig. 3c, *p* = 0.84) NMIBC tissue compared to its normal tissue; to maintain the U:G arrangement, initial experiments were performed with 8-oxoG:G substrates. When looking at each patient individually, four of the high-grade NMIBC tissues showed no change in 8-oxoG incision activity (1.03 ± 0.01 fold) relative to the comparative normal tissue. Three of the high-grade NMIBC tissues did, however, display a small (1.48 ± 0.25 fold) increase in 8-oxoG incision activity, while three of the low-grade NMIBC tissues also exhibited a similar (1.91 ± 0.37) slight increase. In addition, three low-grade and four high-grade NMIBC tissues displayed a decrease in 8-oxoG incision activity compared to normal tissues (2.24 ± 0.91 and 1.99 ± 0.57 fold, respectively). All NMIBC tissues at the Ta/T1 stage showed no significant change in 8-oxoG incision activity relative to their normal controls (Fig. 3d, *p* = 0.85). Nine NMIBC and the corresponding normal tissues were also tested for 8-oxoG:C incision activity, as this substrate is more biologically relevant (Fig. 3e,f). As shown, there was no significant change in 8-oxoG:C incision activity in NMIBC tissues compared to the corresponding normal tissue (Fig. 3f, *p* = 0.195). Fold changes in 8-oxoG:G and 8-oxoG:C incision activities of each NMIBC tissue to its corresponding normal tissue are shown in Supplementary Table S1 online. The same blot used for UDG was stripped and analyzed using antibody against OGG1. Strikingly, OGG1 protein level was found dramatically higher (fold range 1.24 to 85) in NMIBC tissues compared to their corresponding normal tissues (Fig. 3g, Supplementary Table S2 online). OGG1 enzyme activity did not correlate significantly with its protein level in NMIBC and the corresponding normal tissue (*p* > 0.05) (Supplementary Table S3 online). As a recent report has suggested that DNA damage binding protein 1 (DDB1), a protein that is a component of the UV-DDB DNA damage recognition complex, exhibits the ability to stimulate OGG1 activity^43^, we examined the levels of DDB1 in NMIBC and corresponding normal tissue extracts. We observed a significant correlation between the protein levels of DDB1 and OGG1 in NMIBC tissues (r = 0.57, *p* = 0.02) and in the corresponding normal tissues (r = 0.86, *p* = 0.0001) (Supplementary Fig. S3h). The levels of DDB1 do not correlate with 8-oxoG:G or 8-oxoG:C activities (*p* > 0.05) (Supplementary Fig. S3h). Our results suggest that DDB1 is not operating as a major stimulating factor of OGG1, and that the activity of the glycosylase is being suppressed by an unknown mechanism.

### NMIBC tissues have increased AP endonuclease activity

**Figure 4:**
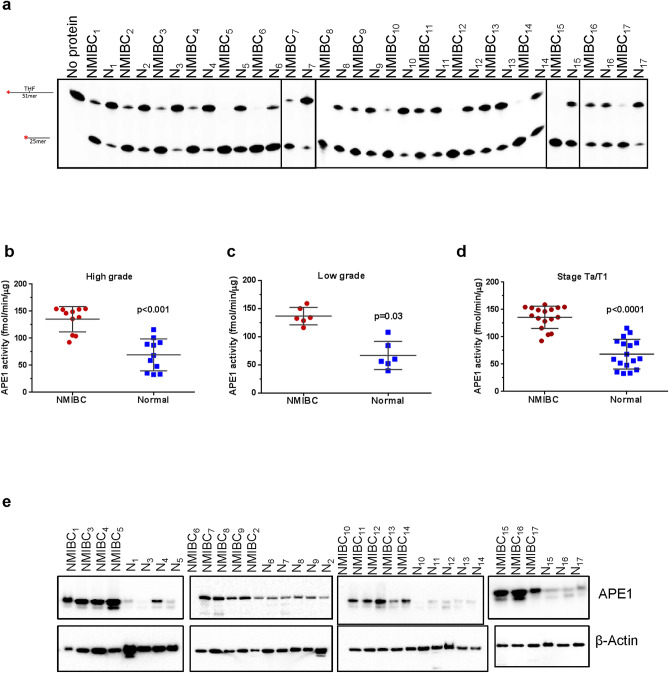
APE1 activity and protein levels in NMIBC and the corresponding normal tissues. (**a**) Representative gel for THF incision in NMIBC and their corresponding normal tissues. Incision products are 25mer derived from cleaved ^32^P-labeled 51mer DNA substrate containing uracil THF at position 26. N, the corresponding normal tissue. Quantitation of THF incision from high-grade (**b**), low-grade (**c**) and Ta/T1 stages (**d**) of all NMIBC and their corresponding normal tissues (fmol/μg/min). (**e**) Western blot analysis of APE1 and β-actin in NMIBC and their corresponding normal tissues. The full-length gels/blots are presented in Supplementary Fig. 4. Radioactive gels were visualized by Typhoon FLA 9500 PhosphorImager and analyzed using the ImageQuant software TL v8.1 (https://www.cytivalifesciences.com/en/us/shop/protein-analysis/molecular-imaging-for-proteins/imaging-software/imagequant-tl-8-1-p-00110).

We next measured AP site incision activity, perhaps exclusively carried out by APE1, in whole tissue extracts of NMIBC and corresponding normal tissues using a tetrahydrofuran (THF)-containing DNA substrate. THF is analog of an AP site that can be cleaved by APE1, a class II AP endonuclease, but not bifunctional DNA glycosylases that use alternative incision mechanisms^44^. A representative gel for AP endonuclease (from hereon APE1) activity using whole tissue extracts is shown in Fig. 4a. As with the other substrates, a lesion-free oligodeoxynucleotide duplex was used as a negative control, and no product was observed (data not shown). APE1 activity was found significantly higher in both high-grade (Fig. 4b, *p* < 0.001) and low-grade (Fig. 4c, *p* = 0.03) NMIBC tissues compared with the corresponding normal tissue. The activity was 2.15 ± 0.35 and 2.35 ± 0.35 fold higher in low- and high-grade NMIBC tissues, respectively. Analyzing all NMIBC patients (low and high grades) at the Ta/T1 stage revealed a significant increase in APE1 activity (*p* < 0.0001) in NMIBC compared to normal tissues (Fig. 4d, Supplementary Table S1 online). Furthermore, APE1 protein level was at least 1.86 fold higher in NMBIC tissues than their corresponding normal tissues (Fig. 4e, Supplementary Table S2 online). APE1 protein levels were correlated significantly with the activity levels of APE1 in NMIBC and the corresponding normal tissues (r = 0.49, *p* = 0.05 and r = 0.48, *p* = 0.05) (Supplementary Table S3 online).

### NMIBC tissues have increased gap-filling activity

**Figure 5:**
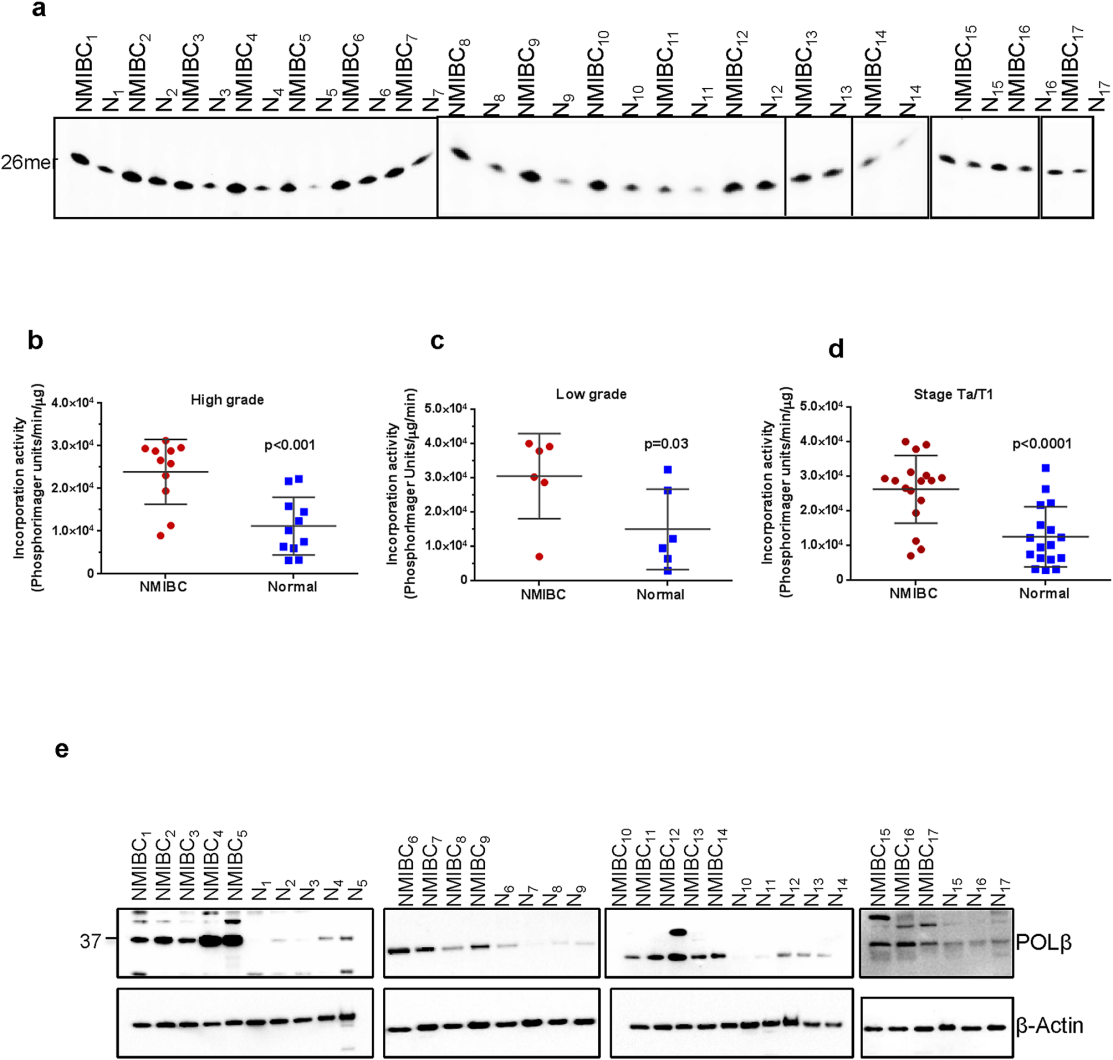
Single nucleotide gap-filling activity and POLβ protein levels in NMIBC and their corresponding normal tissues. (**a**) Representative gel for single nucleotide gap-filling activity showing products of ^32^P-dCTP incorporation (N, the corresponding normal tissue). Single nucleotide gap-filling activity values of high-grade (**b**), low-grade (**c**) and Ta/T1 stages (**d**) of all NMIBC and the corresponding normal tissues. (**e**) Western blot of POLβ and β-actin in NMIBC and their corresponding normal tissue extracts. Low-grade sample numbers are 2, 4, 9–12; high-grade sample numbers are 1, 3, 5–8, 13–17. The full-length gels/blots are presented in Supplementary Fig. 5. Radioactive gels were visualized by Typhoon FLA 9500 PhosphorImager and analyzed using the ImageQuant software TL v8.1 (https://www.cytivalifesciences.com/en/us/shop/protein-analysis/molecular-imaging-for-proteins/imaging-software/imagequant-tl-8-1-p-00110).

Proceeding through the BER pathway, we next analyzed 1-nt gap-filling activity in NMIBC and the corresponding normal tissues, monitoring incorporation of a radiolabeled dCTP nucleotide into a 51mer duplex harboring a 1-nt gap at position 26 (Table 2). A representative gel for 1-nt gap filling is shown in Fig. 5a. Quantification of the results revealed that single nucleotide gap-filling activity in both low- and high-grade NMIBC tissues was significantly elevated in comparison to the corresponding normal tissue (*p* = 0.03 and *p* = 0.001, respectively) (Fig. 5b,c). The gap-filling activity was 2.650 ± 0.51 and 2.77 ± 0.52 fold higher in low- and high-grade NMBIC tissues, respectively (Supplementary Table S1 online). Analyzing all NMIBC patients at Ta/T1 stage demonstrated a significant increase in 1-nt nucleotide gap-filling activity (*p* < 0.0001) in cancer tissues relative to normal material (Fig. 5d). Since POLβ is the predominant protein to carry out single nucleotide gap-filling ^6,53^, we measured POLβ levels and found them to be at least 1.86 fold higher in NMBIC tissues than the corresponding normal tissues (Fig. 5e, Supplementary Table S2 online). POLβ protein levels were significantly correlated with the activity levels of gap-filling in the corresponding normal tissues (r = 0.0.57, *p* = 0.02) but not in NMIBC tissues (r = 0.32, *p* = 0.21) (Supplementary Table S3 online).

### Uracil-initiated total BER activity correlates with uracil incision activity

Several studies have reported different rate-limiting factors in BER, including UDG, APE1, POLβ or the lyase step, with investigators arguing that the rate-limiting step may change with age, cell and lesion type. Knowledge of the rate-limiting step in BER would provide important insight into determining overall BER capacity^45–49^. In this study, uracil-initiated total BER activity (incorporation plus ligation) positively and significantly correlates with uracil incision activity in both NMIBC and the corresponding normal tissue (Table 3). Conversely, uracil-initiated total BER activity is not significantly correlated with APE1 activity or single nucleotide gap filling (Table 3). Thus, uracil incision activity (i.e., DNA glycosylase activity) appears to be rate-limiting in BER, at least for this lesion, in NMIBC and normal bladder tissues.

**Table 3:** Correlation of BER enzyme activity to the activity of uracil-initiated BER in NMIBC and their corresponding normal tissues.

Uracil initiated total BER activity				
		R^2^*	r*	*p* value^†^
Uracil incision	NMIBC	0.42	0.65	0.005
	Normal	0.51	0.72	0.001
APE1 incision	NMIBC	0.15	0.39	0.12
	Normal	0.18	0.42	0.09
Gap filling activity	NMIBC	0.03	0.16	0.54
	Normal	0.03	0.17	0.49

*R^2^ is the Pearson coefficient of determination. *r is the Pearson correlation coefficient. ^†^*p* is a statistical significance of association, two-sided (*p* < 0.05).

### BER protein levels are mostly higher in BC material

**Figure 6:**
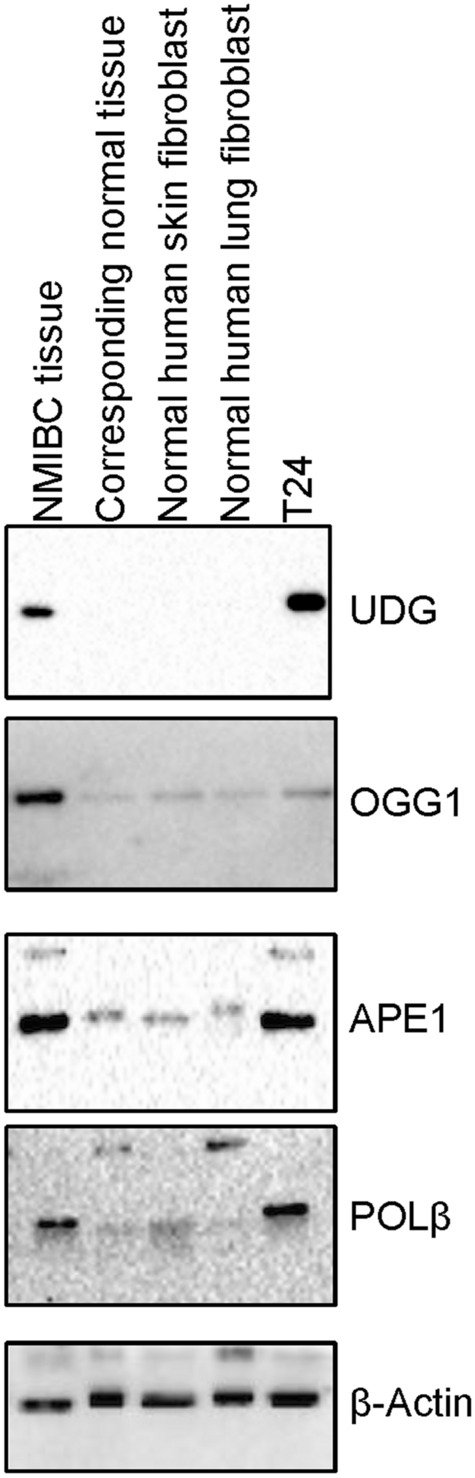
BER protein levels in normal and NMIBC cells/tissues. Western blot analysis of UDG, OGG1, APE1, and POLβ proteins in NMIBC tissue, the corresponding normal tissue (N), normal human skin fibroblast cell line (GM00969), normal human lung fibroblast cell (IMR-90) line, and T24 NMIBC cell line. The full-length gels/blots are presented in Supplementary Fig. 6.

To more broadly compare the BER protein levels in NMIBC tissue, we performed western blotting experiments with extracts from the T24 urinary BC cell line and different types of normal tissues/cells [normal bladder tissue, normal human skin fibroblast cell line (GM00969), and normal human lung fibroblast cell line (IMR-90)]. As shown in Fig. 6, the amount of UDG, OGG1, APE1 and POLβ proteins in NMIBC tissues and BC cells are higher than normal tissue and cells. The amount of UDG protein appears to be very low in normal cells/tissues, as we could not detect the protein via western blot analysis (Fig. 6). The protein levels of NMIBC tissue compared to the corresponding normal tissue are as follows: OGG1 is 7.03 ± 1.02 fold, APE1 is 4.45 ± 0.07 fold, and POLβ is 5.56 ± 0.22 fold higher (Fig. 6). The protein levels of T24 cell line compared to normal human skin fibroblast cell line (GM00969) and normal human lung fibroblast cell line (IMR-90) are as follows: OGG1 is 1.29 ± 0.4 and 0.99 ± 0.00 fold, APE1 is 32.35 ± 1.4 and 5.36 ± 0.06 fold, and POLβ is 120.36 ± 4.1 and 6.82 ± 0.01 fold higher, respectively (Fig. 6).

### BER activities show wide inter-individual variation among NMIBC and normal tissues

**Figure 7:**
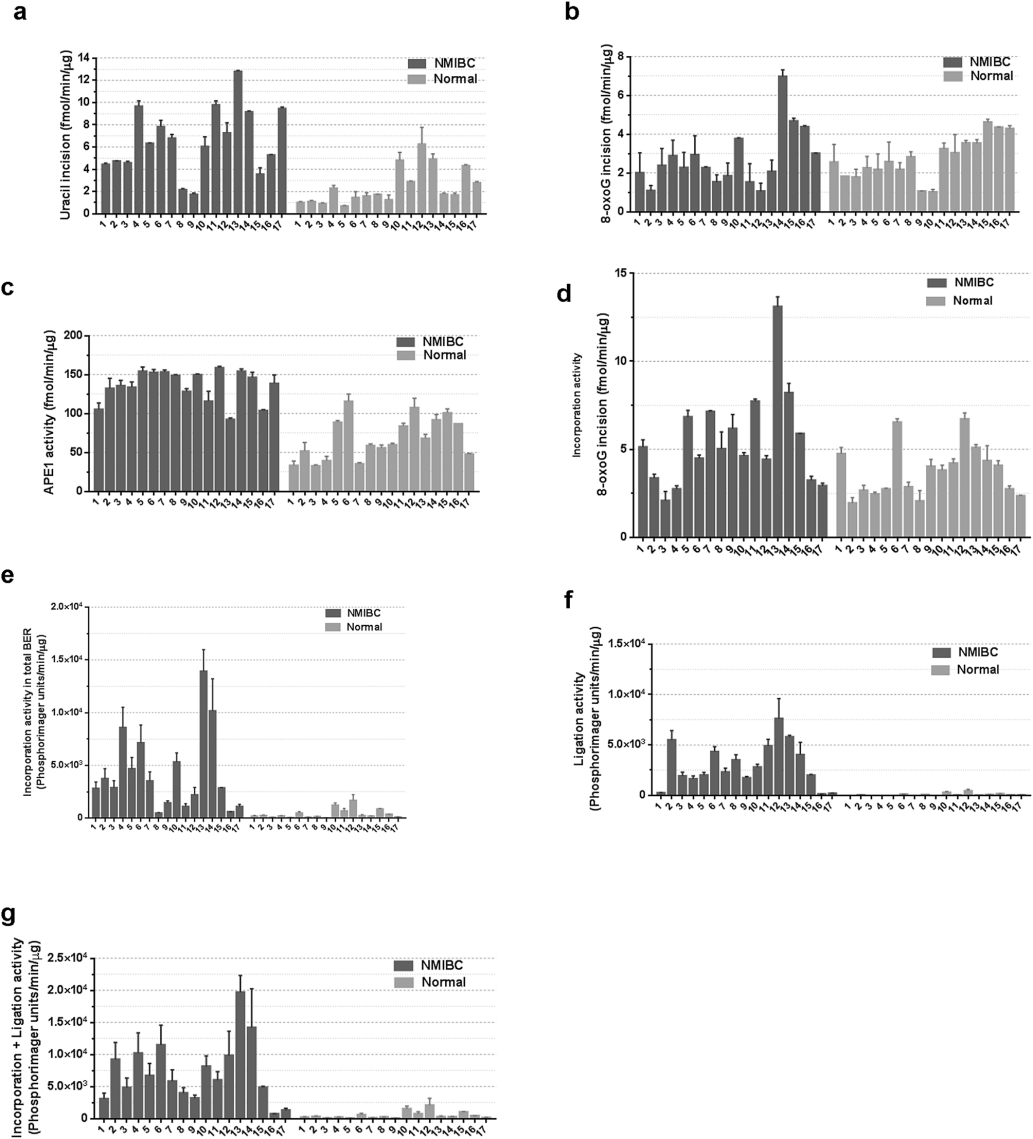
Inter-individual variability in BER of seventeen NMIBC and seventeen corresponding normal tissues. (**a**) Uracil incision activity. (**b**) 8-oxoG incision activity. (**c**) APE1 activity. (**d**) Single nucleotide gap-filling activity. (**e**) Incorporation activity. (**f**) Ligation activity. (**g**) Incorporation plus ligation activity. Low-grade sample numbers are 2, 4, 9–12; high-grade sample numbers are 1, 3, 5–8, 13–17.

Using the repair activity measurements from the studies above, we evaluated the distribution of BER activities among the cancer and corresponding normal tissues obtained from the seventeen NMIBC patients (Fig. 7a-g). Using uracil incision activity as an example, NMIBC tissues exhibited a 7.02 fold inter-individual variability in repair activity (from 1.75 fmol/min/μg for NMIBC_9_ to 12.28 fmol/min/μg for NMIBC_13_), with a mean of 6.32 for NMIBC tissues of all patients (Fig. 7a). The inter-individual variations in NMIBC tissues for the other BER functions are as follows: 5.35 fold in 8-oxoG incision activity (Fig. 7b), 2.58 fold in APE1 activity (Fig. 7c), 9.81 fold in 1-nt gap filling activity (Fig. 7d), and 27.28 fold in incorporation plus ligation BER activity (Fig. 7g). The inter-individual variations in the corresponding healthy tissues are as follows: 8.83 fold in uracil incision activity (Fig. 7a), 4.02 fold in 8-oxoG incision activity (Fig. 7B), 3.53 fold in APE1 activity (Fig. 7c), 8.83 fold in 1-nt gap filling activity (Fig. 7d), and 53.94 fold in incorporation plus ligation activity (Fig. 7g).

## Discussion

Because alterations in BER capacity of various cancer types can influence cancer susceptibility, recurrence, progression, and responses to chemotherapy and radiotherapy^6^, we have investigated BER activities in NMIBC tissue. Previous studies have suggested the importance of some BER enzymes in BC progression and the response to oxidative stress caused by BCG^16,22,23^. To the best of our knowledge, this is the first study demonstrating that NMIBC tissues have increased total BER activity in comparison to normal bladder tissue from the exact same patient. The consistently enhanced BER capacity of NMIBC could play an important role in any number of aspects of carcinogenesis, including preserving the genome integrity in the primary tumor environment to facilitate progression and metastasis with increased efficacy^50^.

We observed a significant increase in uracil (U:G and ssU) incision activity and UDG protein levels in both low- and high-grade NMIBC compared to the corresponding normal tissues. Enhanced uracil (U:G) incision activity and UDG protein levels have also been reported in human colorectal cancer (CRC) tissues compared to adjacent normal mucosa^51^. Notably, UDG expression is cell cycle regulated, with the highest level of UDG being observed in S phase^42,51^, seemingly tying its production to replicative status. In addition, overexpression of *UNG* in non-small cell lung cancer (NSCLC) tissues and lung cancer cell lines is associated with a more aggressive phenotype^52,53^. It has been speculated that cancer tissues have a higher demand for UDG activity due to their high proliferation rate and increased synthesis of the precursor nucleotides that result in enhanced uracil incorporation into DNA^51^. Indeed, Pulukuri et al.^54^ have demonstrated that the function of UDG is essential for cancer cell survival. Moreover, UDG deficiency in cancer cells, including colon and lung cancers, results in sensitivity to chemotherapeutic agents that introduce uracil, 5-fluorouracil (5-FU), and 5-fluorodeoxyuridine lesions into DNA^52,53,55–58^. Thus, the enhanced UDG activity could be necessary for NMIBC survival and provide resistance to chemotherapeutic agents that induce DNA lesions repaired by UDG.

Collectively, studies indicate that UDG expression or activity levels do not simply correlate with the cytotoxicity of 5-FU. In particular, some studies have shown that UDG levels do not influence the cytotoxicity of 5-FU, whereas others have shown that high UDG levels cause cellular lethality to 5-FU due to futile BER cycling^59^. In the current study, while there is not much difference in UDG protein levels in T24 BC cells compared to SV-40 transformed SV-HUC-1 normal bladder cells (1.2 fold higher in T24), T24 cells are almost 50-fold more sensitive to 5-FU treatment (100 μM at 72 h post-treatment) (Supplementary Fig. S7). Thus, the cellular lethality of 5-FU depends on many factors, including BER components, such as UDG, SMUG1 and APE1, and the mismatch repair pathway, with several mechanistic details still requiring further elucidation^59^.

Visnes et al.^49^ observed a correlation between activity and protein levels of UNG2, the nuclear form of UDG, in nuclear extracts of different cancer cell lines. We used whole tissue extracts in the current study, and the UDG protein level also correlated with uracil incision activity in whole NMIBC tissue extracts. Nevertheless, a limitation of the present study is the lack of ability to address issues of subcellular localization of the different proteins, a factor that has been shown to be significantly affected by transformation and the cancer state. Future analysis should aim to conduct immunohistochemistry to examine BER protein localization in different BC stages.

OGG1 is the main DNA glycosylase for the repair of one of the most common and mutagenic oxidative DNA lesions, 8-oxoG. If it is not repaired, 8-oxoG can induce GC to TA transversions, consistent with the accumulation of 8-oxoG increasing mutagenesis and the risk of carcinogenesis. OGG1 activity was found lower in leukocytes and lung tumor tissues of NSCLC^60–63^, and leukocytes of squamous cell carcinoma of head and neck (SCCHN) cancers^64^. The reduced OGG1 activity, and in turn, a high amount of 8-oxoG is associated with an increased risk of NSCLC and SCCHN^60–64^. In contrast, OGG1 activity was found significantly higher in leukocytes and tumor tissues of CRC patients^65,66^. It has been suggested that increased oxidative stress in CRC stimulates the expression and activity of OGG1, which at its normal level is not sufficient to counteract the increased oxidative DNA damage^65^. Paz-Elizur et al.^62,64,67–69^ demonstrated that the presence of cancer did not cause low OGG1 activity, but instead the low activity represents an inherent characteristic of SCCHN individuals, putting them at risk of cancer development. We found that seven of NMIBC tissues have reduced (1.96 ± 0.33 fold) 8-oxoG incision activity, whereas six of them had increased (1.86 ± 0.45 fold) and four of them had similar activities compared to normal tissue. Furthermore, in line with other studies^61,68–71^, we did not find a significant correlation between OGG1 activity and its protein levels in NMIBC and the corresponding normal tissues. It has been suggested that OGG1 activity can be affected by several other factors, including BER enzymes, such as XRCC1, APE1^61,72,73^. Thus, mRNA expression and the protein levels of OGG1 cannot be used to predict OGG1 activity^61,69^. Notably, we observed marked increases in OGG1 protein levels in NMIBC tissue relative to the normal material (that did not correlate with the 8-oxoG incision measurements), suggesting that much of the protein produced is likely inactive or inactivated, possibly through post-translational mechanisms. In addition, OGG1 activity can be influenced by many other factors. Recently, it has been shown that UV-DDB stimulates OGG1 incision activity^43^. Notably, a significant correlation was observed between the protein levels of DDB1 and OGG1 in both NMIBC and in the corresponding normal tissues, although DDB1 does not appear to be responsible for the lack of correlation between OGG1 protein and activity levels.

APE1 repairs mutagenic and cytotoxic AP sites in DNA. The overexpression of APE1 has been demonstrated in the development, recurrence, and progression of different cancer types, including BC, and in resistance to various anticancer drugs and ionizing radiation^6,14–27,74,75^. Previous studies demonstrated high expression levels of APE1 in serum, urine, and tissue samples of NMIBC and MIBC patients who had not received chemotherapy or radiotherapy^16,22,23,73^. The increased APE1 levels are associated with bladder tumor stage, grade, muscle invasion, and recurrence. Thus, it has been suggested that APE1 could be a good prognostic biomarker for BC^22,23^. However, these studies reported only gene expression or protein levels of APE1 in serum and urine samples of NMIBC compared to healthy individuals^22,23^, in low-grade NMIBC tissues compared to high-grade tissues^16,22^, and in BC tissue compared to benign urothelium^73^. In this study, we have expanded the analysis to compare NMIBC tissues with their corresponding normal tissues in terms of both total AP endonuclease activity and APE1 protein levels. The results of our work agree with previous findings^16,22,23,73^ that NMIBC tissues have increased APE1 protein and activity levels, supporting the idea that APE1 would be an excellent prognostic biomarker for BC. Inhibition of APE1/Ref1 redox signaling reduces the BC cell proliferation and that enhances the therapeutic effect of cisplatin on BC cells, suggesting that APE1/Ref1 is a good drug target for the treatment of BC^73^.

POLβ is overexpressed in several different types of cancers, including stomach, ovary, colon, prostate, and BC^16,28–31^. Furthermore, the activity and protein levels of POLβ are increased in human ovarian tumor cells^32^. It has been suggested that the overexpression of POLβ may play a role in cancer predisposition and tumor progression, because the polymerase increases the spontaneous mutation rate due to its error-prone nature^32,33,76^. Chantre-Justino et al.^16^ compared POLβ expression levels between low- and high-grade NMIBC tissues, and found that the expression level of POLβ is increased in high-grade compared to the low-grade NMIBC tissues. We showed that gap-filling activity, a major function of POLβ, and POLβ protein levels are significantly higher in both low- and high-grade NMIBC tissues compared to corresponding normal tissue. Collectively, the results suggest that increased POLβ activity is associated with NMIBC development and progression. Interestingly, POLβ protein and gap-filling activity levels are correlated significantly in the corresponding normal tissues, but not in NMIBC tissues. It is possible that not all overexpressed POLβ proteins in NMIBC tissues that we detected on the western blot are active. Several tumors carry mutations in the *POLβ* gene that are not found in the germline. Many of these sporadic *POLβ* gene variants have impaired, often reduced, activity^9^, which may explain the lack of correlation between gap-filling activity and POLβ protein levels.

Visnes et al.^49^ identified UNG2 as the major rate-limiting factor in uracil-initiated total BER in human cancer cell lines. The observed positive and significant correlation between uracil-initiated total BER activity and UDG activity in both NMIBC and normal tissue supports this result. That said, in the Visnes et al.^49^ study, ligase III was also found as a minor rate-regulator for U:G repair, indicating that no single factor can be identified as rate-limiting in human cancer cell lines^49^. Furthermore, previous studies have demonstrated that the dRP lyase activity of POLβ or nick ligation is the rate-limiting step in BER^11^. Brenerman et al.^11^ suggested that because of the variability in the rate of repair among individuals, any step of BER could be rate-limiting. The inter-individual variations in the activities of BER enzymes may affect the correlation between the activities of total BER and specific BER enzymes.

Poly-ADP ribosylation (PARylation) by PARP1/2 plays a key role in various cellular processes, such as transcription, DNA damage response and apoptosis. PARylation increases the recruitment of BER proteins to the DNA damage site^77^. NMIBC tissues have higher levels of both PARylation and BER capacity compared with the corresponding normal tissues (Supplementary Fig. S8). A general increase in BER capacity would presumably protect the cell from genotoxic stress. Additionally, elevated BER could provide the cell with a growth advantage as it converts from a normal to transformed state. However, it is also known that imbalances within the steps of BER, which may arise from up-regulation of only a subset of enzymes, can have more harmful effects than simply reduced pathway capacity. Whether the increased activities reported herein contributed to the early genomic instability that promoted transformation or supported increased growth rates during the carcinogenic process is unknown; but it seems reasonable to conclude that at minimum, the broadly increased BER would play a role in anticancer genotoxin agent resistance.

In conclusion, enhanced BER capacity may play an important role in the etiology and prognosis of NMIBC. Thus, the activity of BER proteins might serve as useful biomarkers in the prognosis and progression of NMIBC or the response to genotoxic therapeutics, such as BCG. Future retrospective and prospective studies are needed to verify that activities of BER enzymes can be used as a biomarker for NMIBC prognosis, progression, and response to genotoxic agents.

## Materials and methods

### NMIBC patients and samples

Seventeen NMIBC tissues and non-cancerous normal bladder tissue from the same individual were obtained using the cold cup biopsy technique during TURBT at the Department of Urology, Acibadem Maslak Hospital, Istanbul, Turkey. The fresh tissue samples were immediately snap-frozen and stored in liquid nitrogen. Evaluation and grading of the NMIBC and corresponding normal tissues were done by the Pathology Department of Acibadem Maslak Hospital according to the previously published criteria^78^. The characteristics of the seventeen NMIBC patients are presented in Table 1. The mean age of the patients was 68.65 ± 15.02 years (range 37 to 92). Of the seventeen NMIBC patients, six were diagnosed as low-grade, and eleven were diagnosed as high-grade. Fourteen NMIBC tissues were Ta stages, and three were T1 stages (Table 1). All NMIBC patients used in this study were newly diagnosed and had not been previously treated with BCG, chemotherapy, or radiotherapy at the time of the biopsy. Written informed consents were obtained from all patients before participation in this study. The study was approved by the Ethics Committee of Acibadem University (ACU) and Acibadem Health Institutions Medical Research (ATADEK), and was performed in accordance with the guidelines of human research. All subjects involved in the study provided their written informed consent according to the Declaration of Helsinki, as approved by the Ethics Committee of ACU and ATADEK.

### Whole tissue lysates preparation for BER assays

Whole tissue lysates were prepared as previously described^79^ with some modifications. Briefly, tissues were suspended in ice-cold buffer containing 210 mM D-mannitol, 70 mM sucrose, 10 mM HEPES, 1 mM EGTA, 2 mM EDTA, 0.75 mM spermidine, 0.15 mM spermine, 5 mM DTT, 1X complete protease inhibitor tablet (Roche Applied Sciences, USA) and homogenized using a Dounce glass-glass homogenizer. The homogenate was spun at 16,000xg for 15 min at 4 °C. The pellet was resuspended in one volume of buffer 1 (10 mM Tris–HCl, pH 7.8 and 200 mM KCl) and two volumes of buffer 2 (10 mM Tris–HCl, pH 7.8, 200 mM KCl, 2 mM EDTA, 40% glycerol, 0.2% NP-40, 2 mM DTT, 1 mM PMSF, 1X complete protease inhibitor tablet) followed by sonication to fragmentize DNA. After incubation for 2 h at 4 °C with rocking, the cell lysate was centrifuged at 130,000xg for 1 h and the supernatant dialyzed overnight in dialysis buffer (25 mM HEPES–KOH, pH 7.5, 100 mM KCl, 1 mM EDTA, 1 mM DTT, 15% glycerol). Subsequently, the samples were snap-frozen in liquid nitrogen and stored at − 80 °C. Protein concentration was determined using the Bio-Rad protein assay (Bio-Rad, USA). Besides, β-actin levels were determined by the western blot analysis in NMIBC and corresponding normal tissues as described in the western blot section.

### Oligodeoxynucleotides

The sequences of the oligodeoxynucleotides used in this study are shown in Table 2. The oligodeoxynucleotides were purchased from DNA Technology, Denmark and the oligodeoxynucleotide containing C opposite to 8-oxoG was purchased from Eurofins. The oligodeoxynucleotides used in DNA glycosylase and APE1 activity assays were 5′-end-labeled using T4 polynucleotide kinase and [γ-^32^P]ATP (Perkin Elmer, USA) as described before^79^. The oligodeoxynucleotides were then annealed to the complementary strands by heating the samples at 90 °C for 5 min and slowly cooling to room temperature. For single strand uracil incision assay, the single-stranded oligodeoxynucleotide containing uracil (Table 1) protected by phosphorothioate (four bonds) at each end to avoid degradation of the substrate by nucleases in tissue extracts was purchased from Eurofins and 5′-end-labeled using T4 polynucleotide kinase and [γ-^32^P]ATP (Perkin Elmer, USA) as described before^79^.

### Repair synthesis incorporation assay

Reactions were done as previously described^79,80^ with some modifications. Briefly, reactions (10 µl) were done in BER buffer (40 mM HEPES, pH 7.6, 0.1 mM EDTA, 5 mM MgCl_2_, 0.2 mg/ml BSA, 50 mM KCl, 1 mM DTT, 40 mM phosphocreatine, 100 µg/ml creatine phosphokinase (Sigma-Aldrich, USA), 2 mM ATP (GE-Healthcare, USA), 40 µM of each dATP, dTTP, dGTP and 4 µM of dCTP (Roche Applied Sciences, USA), 2 µCi ^32^P-dCTP (Perkin Elmer, USA), 3% glycerol, and 100 fmol of uracil-containing double-stranded DNA substrate). Reactions were initiated by adding 0.4 µg whole tissue lysate and were incubated at 37 °C for 1 h, followed by termination with the equal volume of formamide stop dye (90% formamide, 10 mM EDTA, 0.01% bromphenol blue, 0.01% xylene cyanol). Samples were heated at 37 °C for 10 min and then run on 20% denaturing polyacrylamide gel (PAGE-Urea). The reactions were visualized using a Typhoon FLA 9500 PhosphorImager and quantitated with ImageQuant TL v8.1 software (GE Healthcare). The experiments were performed at least in duplicate. The incorporation, ligation and incorporation plus ligation activities are presented as PhosphorImager units per min per μg protein.

### Activities of DNA glycosylases

Incision activity assays were performed as previously described^49,63,79,80^ with some modifications. Incision of uracil was performed in a reaction mixture (10 µl) containing 70 mM HEPES–KOH, pH 7.4, 5 mM EDTA, 1 mM DTT, 50 mM NaCl, 10% glycerol and 100 fmol of ^32^P-labeled double-stranded uracil-containing DNA substrate (U:G) (Table 2). The reactions were initiated by adding 0.25 µg whole tissue extract or 1 U recombinant UDG protein (New England Biolabs, USA) for uracil incision and incubated at 37 °C for 30 min. UDG was inhibited by pre-incubating tissue extracts with 1 U uracil glycosylase inhibitor (ugi) (New England Biolabs, USA) and with 0.1μg and 1 μg UDG antibody on ice for 10 min (Santa Cruz Biotechnology, USA), followed by uracil incision activity assay as described above. Incision of uracil in ^32^P-labeled single-stranded uracil containing substrate was performed in a reaction mixture (10 µl) containing 40 mM HEPES–KOH, pH 7.8, 1 mM EDTA, 1 mM DTT, 70 mM KCl, 10% glycerol and 100 fmol of ^32^P-labeled single-stranded uracil-containing DNA substrate. The reactions were initiated by adding 0.5 µg whole tissue extract, and incubated at 37 °C for 30 min. The reactions were stopped by the addition of formamide stop dye with 100 mM NaOH. Samples were heated at 95 °C for 10 min and then run on 20% PAGE-Urea gel. The 8-oxoG:G incision activity was performed in 10 µl reaction mixture containing 70 mM HEPES–KOH, pH 7.4, 5 mM EDTA, 1 mM DTT, 75 mM KCl, 10% glycerol and 50 fmol ^32^P-labeled 8-oxoG:G-containing DNA substrate (Table 2). The reaction was initiated by adding 1 µg whole tissue extract and incubated at 37 °C for 60 min. For the complete strand cleavage at the abasic sites, an equal amount of formamide loading dye containing 100 mM NaOH was added and the samples were incubated at 75 °C for 15 min. OGG1 proteins (Trevigen, USA) were used. The 8-oxoG:C incision activity was performed in 20 µl reaction mixture containing 25 mM Tris–HCl pH 7.8, 50 mM NaCl, 5 mM β-mercaptoethanol, 1 mM EDTA, and 50 fmol ^32^P-labeled 8-oxoG:C-containing DNA substrate. The reactions were initiated by adding 1 µg whole tissue extract pre-incubated with 1U ugi as described above, and incubated at 37 °C for 1 h and then incubated with 1 µg proteinase K at 37 °C for 1 h. The reaction mixtures were incubated with 0.2 M NaOH at 70 °C for 30 min, and the reactions were stopped by the addition of formamide stop dye. Samples were incubated at 95 °C for 5 min and resolved by electrophoresis on 20% PAGE-Urea gel. Gels were visualized by Typhoon FLA 9500 PhosphorImager and analyzed using the ImageQuant TL v8.1 software (GE Healthcare, USA). The experiments were performed in triplicate, and the incision activity is presented as fmol of substrate converted to product per min per μg protein.

### AP endonuclease activity

APE1 incision activity was performed as previously described^80,81^ with some modifications. APE1 activity was measured using a 51-mer oligonucleotide containing the abasic site analog THF at position 26 (Table 2). Reactions (10 µl) contained 50 mM HEPES–KOH, pH 7.4, 25 mM KCl, 0.1 mg/ml BSA, 5 mM MgCl_2_, 10% glycerol, 0.01% Tween-20, 50 fmol of ^32^P-labeled THF containing DNA substrate and 0.02 μg whole tissue extract or 1U recombinant APE1 protein (New England Biolabs). Reactions were incubated for 15 min at 37 °C and stopped by the addition of an equal amount of the formamide loading dye, and the samples were heated at 75 °C for 15 min. Samples were separated on 20% PAGE-Urea gel, and gels were visualized by Typhoon FLA 9500 PhosphorImager and analyzed using the ImageQuant software TL v8.1 (GE Healthcare). The experiments were performed in triplicate, and the incision activity is presented as fmol of substrate converted to product per min per μg protein.

### Gap-filling assay

Reactions were done as previously described^80,82^ with some modifications. Single nucleotide gap-filling reactions were performed in 10 µl reaction buffer (40 mM Tris–HCl, pH 7.4, 5 mM MgCl_2_, 50 mM KCl, 1 mM DTT, 5 µM dCTP, 5% glycerol) and contained 100 fmol of duplex oligonucleotide containing 1-nt gap at position 26 (Table 2) and 2 µCi of ^32^P-dCTP (GE Healthcare, USA). Reactions were initiated by adding 0.5 µg whole tissue extract and were incubated at 37 °C for 1 h. After the addition of the equal volume of the formamide stop dye, reactions were incubated at 75 °C for 10 min. Samples were run on 20% PAGE-Urea gel and visualized as described above. The experiments were performed at least in duplicate. The incorporation of ^32^P-dCTP activity was quantified as the increase in the signal intensity. The activity is presented as PhosphorImager units per min per μg protein.

### Western blot analysis

The normal human skin fibroblast cell line (GM00969) was obtained from Coriell Institute for medical research, and the normal human lung fibroblast cell line (IMR-90), and human urinary bladder transitional cell carcinoma cell line (T24), and SV-HUC-1 transformed normal bladder cell line were obtained from the American Type Culture Collection (ATCC). The cell extracts were prepared using radioimmunoprecipitation assay (RIPA) buffer, and protein concentration was determined using the Bio-Rad protein assay (Bio-Rad, USA). Proteins were separated on 4–12% Bis–Tris–plus gels (Invitrogen, USA) and transferred to polyvinylidene fluoride (PVDF) membrane (Bio-Rad, USA). The membrane was blocked in 5% non-fat dry milk (Bio-Rad, USA) with TBST (10 mM Tris–HCl, pH 8.0, 150 mM NaCl, 0.1% Tween-20) followed by exposure to the primary antibody. After incubating with secondary antibody, the membrane was exposed to Pierce ECL Plus or Super Signal West Femto Maximum Sensitivity Substrate according to the manufacturer's protocol (Pierce, USA). The immunoblots were then visualized using ChemiDoc MP Imaging systems (Bio-Rad Laboratories, USA). The immunoblots were analyzed with the following primary antibodies: rabbit anti-APE1 (1:1000, Abcam); mouse anti-POLβ (1:1000, Abcam); mouse anti-UDG (1:500, Santa Cruz Biotechnology); mouse anti-OGG1 (1:500, Santa Cruz Biotechnology), Rabbit anti-UV-DDB1 (1:1000, Cell Signaling Technology), Mouse anti-PAR monoclonal antibody (1:1000, Trevigen) and mouse anti-beta-actin (1:1000; Cell Signaling). PAR-PARP western control protein (Trevigen). Secondary antibodies employed: anti-mouse IgG, HRP linked (1:5000, Cell Signaling) and anti-rabbit IgG, HRP linked (1:5000, Cell Signaling). The membranes were stripped using restore plus western blot stripping buffer according to the manufacturer's protocol (Thermo Scientific, USA) to re-probe with different antibodies.

### Statistical analysis

Statistical analysis was performed using the GraphPad Prism 6.1 software. Differences in the activities between NMIBC tissue and the corresponding normal tissue from the same individuals were analyzed using the Wilcoxon matched-pairs signed-rank test. The Pearson test was performed for correlation analysis. To use Pearson`s correlation test, all data were examined for normality using D'Agostino-Pearson omnibus test as recommended in GraphPad Prism software, and then the variables that were not normally distributed were normalized via transformation to natural logs. The Pearson correlation coefficient (parametric) measures the strength of the linear relationship between normally distributed variables. Spearman nonparametric correlation makes no assumption about the distribution of the values, as the calculations are based on ranks, not the actual values. *p* < 0.05 was considered statistically significant. All statistical tests were two-sided.

## Supplementary information

Supplementary file1

## Data Availability

All data of this study are available from the corresponding author upon reasonable request.
