# Supplementary material for: Non-muscle invasive bladder cancer tissues have increased base excision repair capacity
Source: Sci Rep. 2020 Oct 1;10:16371. doi: 10.1038/s41598-020-73370-z (PMC7529820; doi:10.1038/s41598-020-73370-z)
Supplement: Supplementary file 1 — Supplementary file1 [file 41598_2020_73370_MOESM1_ESM.pdf]

Supplementary Information

Non-muscle invasive bladder cancer tissues have increased base excision repair capacity

Berna Somuncu[1#], Selcuk Keskin[2#], Fatma Merve Antmen[1], Yesim Saglican[3], Aysegul Ekmekcioglu[1], Tugce Ertuzun[1], Mustafa Bilal Tuna[4], Can Obek[5], David M Wilson III[6], Umit Ince[3], Ali Riza Kural[2], Meltem Muftuoglu[1,7*]

[1]Department of Medical Biotechnology, [2]Department of Urology, [3]Department of Pathology, Acibadem Mehmet Ali Aydinlar University, 34752 Istanbul, Turkey
[4]Department of Urology, Acibadem Maslak Hospital, Istanbul, Turkey
[5]Department of Urology, Acibadem Taksim Hospital, Istanbul, Turkey
[6]Biomedical Research Institute, Hasselt University, 3590 Diepenbeek, Belgium
[7]Department of Molecular Biology and Genetics, Acibadem Mehmet Ali Aydinlar University, 34752 Istanbul, Turkey

[#]Both authors contributed equally to this work.

***Corresponding author**
Meltem Muftuoglu, PhD
Acibadem Mehmet Ali Aydinlar University
Department of Medical Biotechnology
Department of Molecular Biology and Genetics
Kayisdagi Street, No.32, 34752, Atasehir, Istanbul, Turkey
Phone: +90 216 500 4131
E-mail: meltem.muftuoglu@acibadem.edu.tr

This file includes:
Supplementary Tables 1-3
Supplementary Figures 1-8

Supplementary Table S1. Fold changes in total BER, uracil incision, 8-oxoG incision, APE1, and 1-nt gap filling activities. No, number of tissues; FC, Average fold change ± standard deviation; ND, not determined. It is ranged from lowest fold change to highest.

	Total BER FC		Total BER FC		Total BER FC		Uracil incision FC		8-oxoG:G incision FC	8-oxoG:C incision FC		APE1 activity FC		1-nt gap filling FC
No	**Incorporation**	No	**Ligation**	No	**Incorporation plus ligation**	No		No			No		No	
	**Low grade**		**Low grade**		**Low grade**		**Low grade**		**Low grade**	**Low grade**		**Low grade**		**Low grade**
12	1.33±0.03	4	ND	12	4.91±0.70	12	1.19±0.17	12	0.38±0.03	0.62±0.00	11	1.38±0.21	12	1.21±0.01
11	1.67±0.26	10	10.55±3.25	10	5.52±0.58	10	1.26±0.02	2	0.60±0.15	0.80±0.01	9	1.39±1.05	2	1.52±0.02
10	4.43±0.19	12	20.39±6.14	11	8.95±2.43	9	1.49±0.48	11	0.72±0.00	-	12	1.48±0.14	11	2.38±0.02
2	16.57±1.98	2	100.27±9.88	2	33.03±4.13	11	3.39±0.14	4	1.28±0.02	1.30±0.02	10	2.50±0.09	10	3.00±0.24
4	44.11±0.41	9	116.83±24.35	4	52.47±0.13	4	4.25±0.26	9	1.37±0.01	-	2	2.67±0.80	4	3.10±0.16
9	70.61±28.88	11	207.90±83.41	9	89.47±30.12	2	4.34±0.36	10	4.06±0.85	-	4	3.44±0.62	9	4.68±0.15
	**High grade**		**High grade**		**High grade**		**High grade**		**High grade**	**High grade**		**High grade**		**High grade**
16	1.68±0.05	1	ND	16	1.81±0.00	16	1.23±0.02	8	0.46±0.34	0.57±0.02	16	1.19±0.01	13	1.23±0.01
8	3.17±0.07	3	ND	15	4.84±0.30	8	1.25±0.04	13	0.67±0.06	0.72±0.05	6	1.33±0.13	6	1.33±0.02
15	3.31±0.19	5	ND	17	8.83±0.55	15	2.12±0.52	17	0.70±0.02	0.75±0.06	13	1.36±0.11	17	1.51±0.01
17	10.89±2.91	7	ND	8	18.24±1.42	13	2.62±0.24	1	0.76±0.12	-	15	1.45±0.09	7	1.85±0.12
1	17.54±5.78	16	2.97±0.53	3	19.20±6.67	17	3.41±0.10	16	1.00±0.01	1.00±0.08	14	1.68±0.10	1	2.15±0.14
6	15.91±2.39	17	4.71±3.04	6	21.07±4.20	1	4.37±0.34	15	1.01±0.00	-	5	1.74±0.06	8	2.33±0.11
7	39.10±3.05	15	14.44±2.09	14	55.97±6.26	7	4.33±0.61	5	1.05±0.04	1.13±0.04	8	2.53±0.11	14	2.71±0.05
3	43.85±11.72	6	43.14±9.96	7	65.02±2.08	3	4.97±0.37	7	1.05±0.15	1.16±0.05	17	2.89±0.26	3	2.83±0.06
14	53.94±9.25	8	49.88±6.97	3	73.08±20.38	14	5.20±0.27	6	1.16±0.06	-	1	3.18±0.37	15	3.04±0.08
5	73.65±8.20	14	63.31±4.43	5	105.70±7.37	6	5.79±2.44	3	1.31±0.17	-	3	4.16±0.30	16	4.28±0.08
13	87.72±57.39	13	338.19±83.60	13	112.96±74.76	5	8.96±0.33	14	1.97±0.01	-	7	4.31±0.06	5	7.20±0.00

Supplementary Table S2. BER protein levels (BER protein/β-actin). Fold changes (FC; NMIBC/N) in NMIBC and their corresponding normal tissues (N) were detected by Western blot. UN, unmeasurable. It is ranged from lowest fold change to highest.

	UDG				OGG1				APE1				POLβ		
No	NMIBC	N	FC	No	NMIBC	N	FC	No	NMIBC	N	FC	No	NMIBC	N	FC
**Low grade**				**Low grade**				**Low grade**				**Low grade**			
10	0.65±0.05	UN	UN	11	1.61 ±0.12	0.72±0.02	2.24	9	0.32±0.039	0.14±0.05	2.29	12	2.08±0.01	0.41±0.10	5.07
11	0.50±0.02	UN	UN	2	1.22±0.10	0.50±0.05	2.44	11	1.33±0.30	0.25±0.01	5.32	11	1.19±0.20	0.12±0.00	9.92
2	0.57±0.09	0.16±0.02	3.56	10	1.39±0.09	0.27±0.01	5.15	4	0.98±0.05	0.17±0.01	5.76	9	0.68±0.02	0.04±0.009	17.00
4	1.35±0.22	0.28±0.01	4.82	12	1.37±0.26	0.21±0.01	6.52	2	0.60±0.03	0.09±0.006	6.67	2	0.76±0.10	0.02±0.001	38.00
9	1.27±0.17	0.05±0.007	25.40	9	1.40 ±0.10	0.21±0.00	6.67	12	2.46±0.61	0.20±0.01	12.30	4	1.64±0.34	0.04±0.002	41.00
12	1.07±0.13	0.03±0.001	35.67	4	0.72 ±0.03	0.01±0.00	72.00	10	1.51±0.17	0.04±0.00	37.75	10	0.83±0.01	0.01±0.00	83.00
**High grade**				**High grade**				**High grade**				**High grade**			
6	1.65±0.39	0.45±0.03	3.67	6	0.97±0.04	0.78±0.02	1.24	6	0.52±0.6	0.28±0.02	1.86	1	0.31±0..02	UN	UN
8	0.75±0.08	0.12±0.02	6.25	13	0.37±0.06	0.16±0.01	2.31	8	0.26±0.004	0.11±0.002	2.36	13	1.01±0.02	0.55±0.12	1.84
7	1.45±0.40	0.16±0.03	9.06	3	0.94±0.11	0.31±0.08	3.03	7	0.30±0.05	0.11±0.004	2.73	17	0.59±0.03	0.27±0.00	2.19
1	1.42±0.1	0.09±0.01	15.78	8	1.03±0.01	0.25±0.00	4.12	17	2.57±0.61	0.76±0.02	3.38	14	1.01±0.21	0.46±0.00	2.20
13	0.89±0.02	0.05±0.002	17.80	1	1.20±0.02	0.22±0.01	5.45	14	1.16±0.06	0.33±0.02	3.52	16	0.92±0.05	0.16±0.00	5.75
14	1.55±0.21	0.03±0.001	51.67	7	0.66 ±0.02	0.12±0.01	5.50	13	0.93±0.11	0.25±0.01	3.72	15	0.84±0.01	0.13±0.00	6.46
15	0.54±0.10	0.01±0.00	54.00	14	0.57±0.04	0.09±0.01	6.33	15	6.04±0.19	0.74±0.02	8.16	3	0.16±0.01	002±0.001	8.00
16	1.69±0.07	0.03±0.005	56.33	16	0.19 ±0.01	0.02±0.00	9.50	16	9.21±0.17	0.82±0.02	11.23	7	0.34±0.01	0.04±0.00	8.50
3	0.70±0.12	0.01±0.00	70.00	15	1.68±0.05	0.07±0.01	24.00	5	1.45±0.05	0.07±0.001	20.71	8	1.01±0.09	0.05±0.00	20.20
17	0.90±0.07	0.01±0.00	90.00	17	1.14±0.02	0.02±0.00	57.00	3	0.63±0.02	0.03±0.00	21.00	5	1.26±0.25	0.05±0.001	25.20
5	1.34±0.17	0.01±0.00	134.00	5	0.85±0.18	0.01±0.00	85.00	1	1.31±0.17	0.03±0.008	43.67	6	0.87±0.01	0.02±0.001	43.50

Supplementary Table S3. Correlation between BER enzyme activity and its protein level in NMIBC and the corresponding normal tissue. †p is a statistical significance of association, two-sided ($p<0.05$). Bold texts are $p<0.05$.

Protein	BER enzyme activity							
	Pearson correlation coefficients		Spearman correlation coefficients		Pearson correlation coefficients		Spearman correlation coefficients	
	NMIBC		NMIBC		Normal		Normal	
	**r**	**p value†**	**r**	**p value†**	**r**	**p value†**	**r**	**p value†**
UDG	**0.51**	**0.04**	0.34	0.19	-0.37	0.18	-0.18	0.51
OGG1	-0.37	0.14	-0.37	0.14	-0.13	0.61	-0.24	0.34
APE1	**0.49**	**0.05**	**0.58**	**0.02**	**0.48**	**0.05**	**0.59**	**0.02**
POLβ	0.32	0.21	0.15	0.56	**0.57**	**0.02**	0.16	0.53

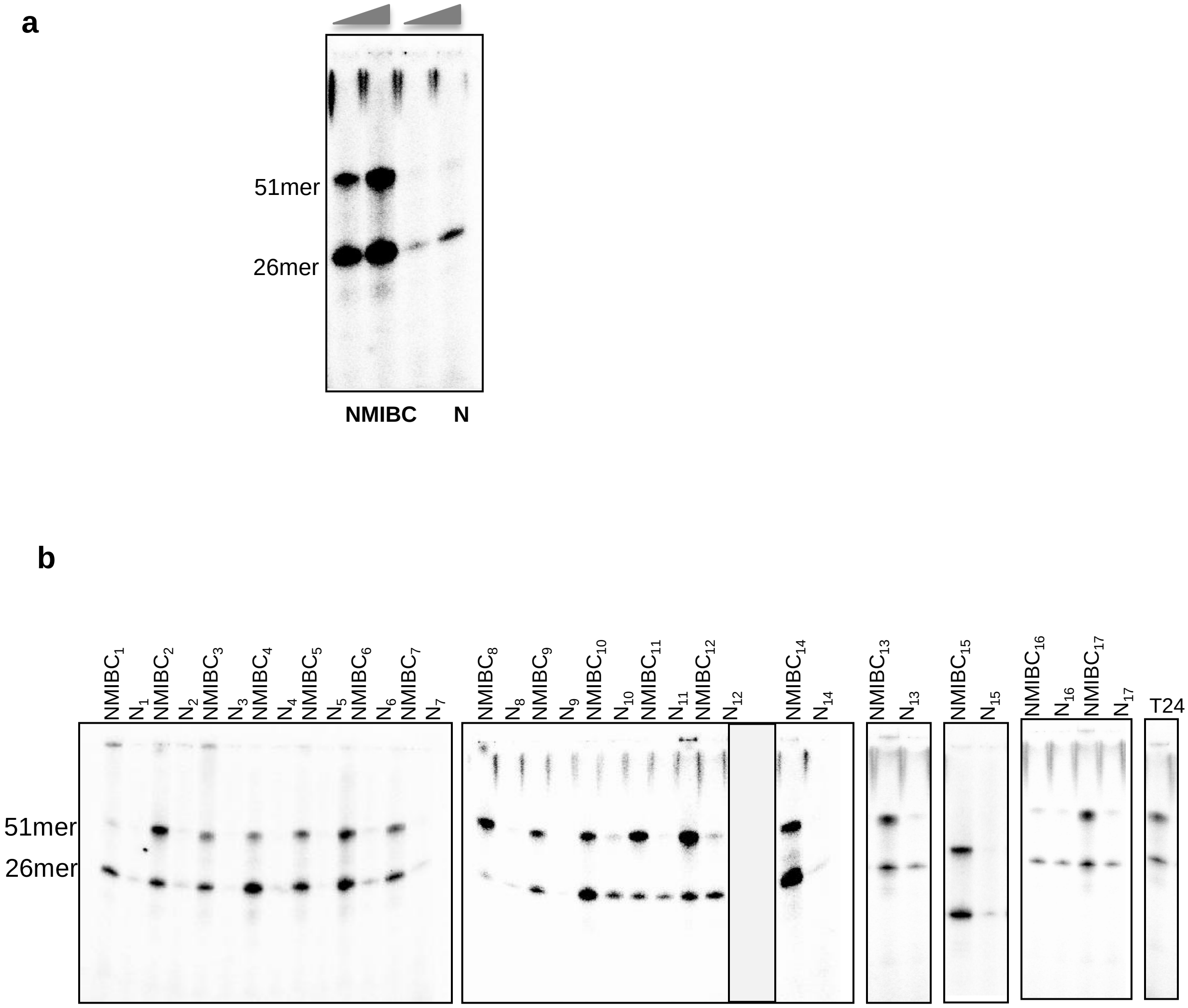

Supplementary Figure 1. Uracil-initiated total BER activity in NMIBC and their corresponding normal tissues. a Total BER activity using increasing concentrations of whole tissue extracts (0.25 µg and 0.5 µg) of NMIBC (lanes 1 and 2) and the corresponding normal tissue (N; lanes 3 and 4). **b** Full length gels for uracil-initiated total BER activity in NMIBC and N tissues and T24 cells.

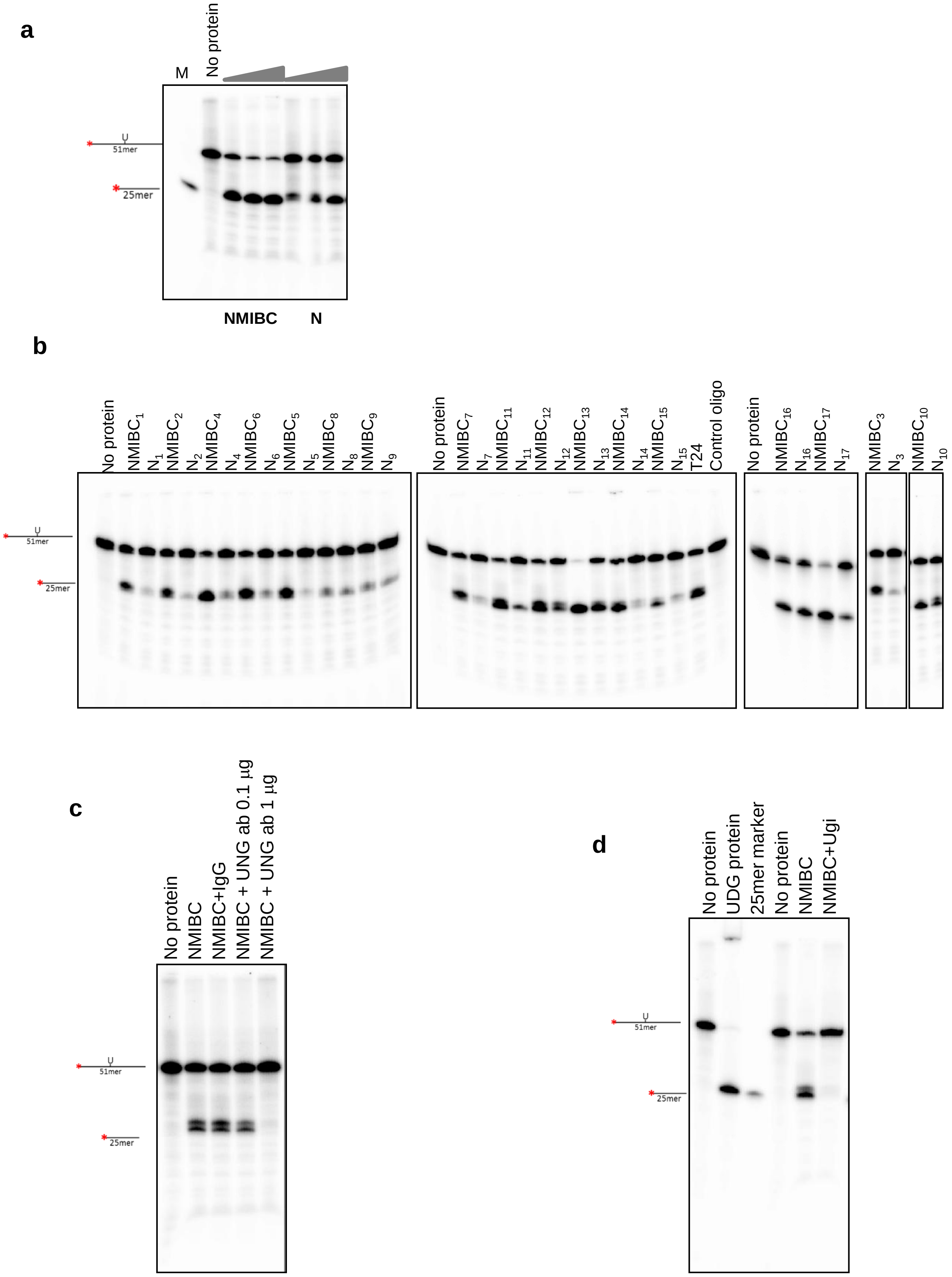

Supplementary Figure 2

e

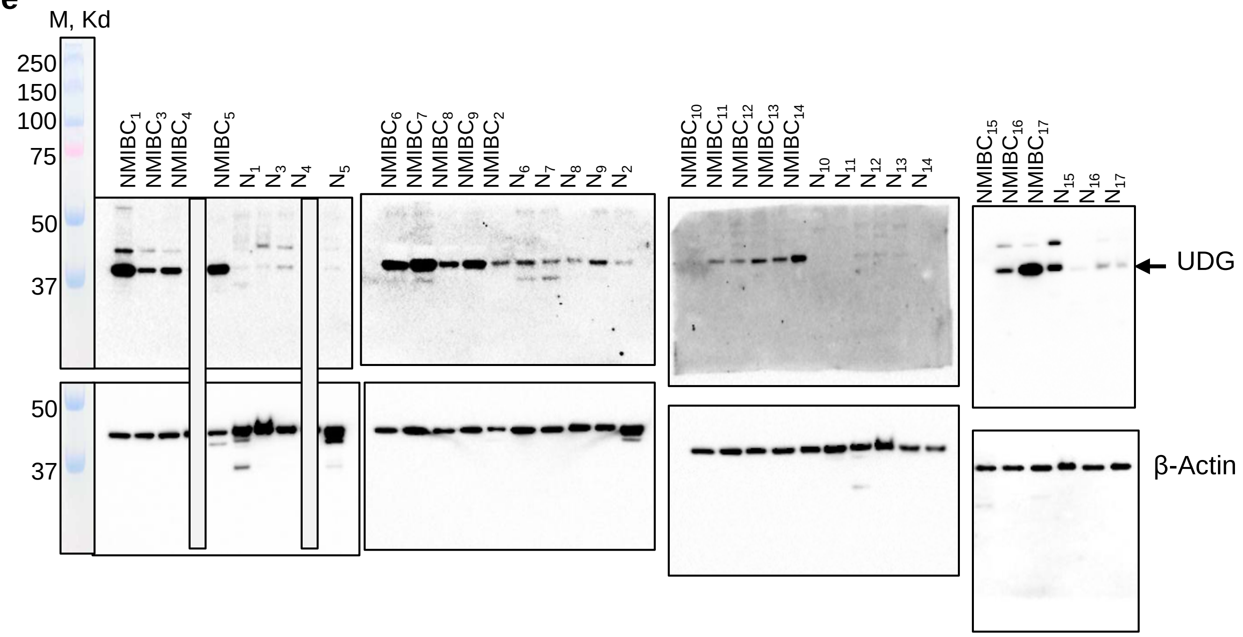

f

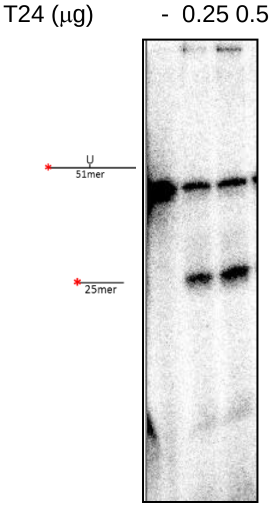

g

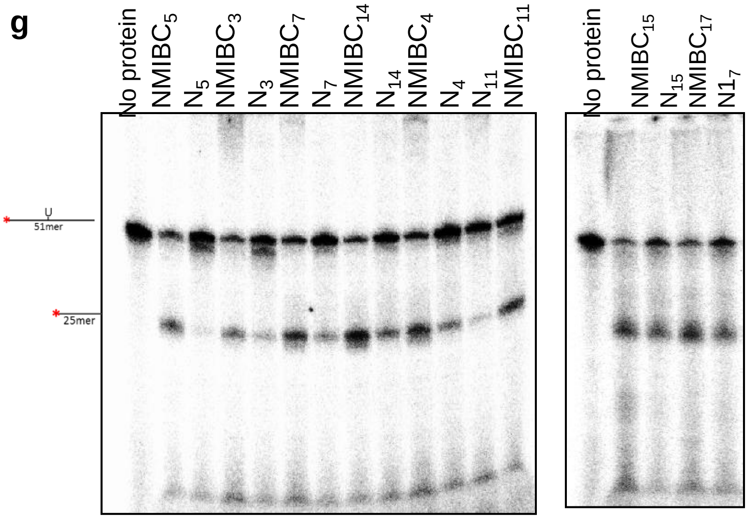

Supplementary Figure 2. Uracil incision activities and UDG protein levels in NMIBC and their corresponding normal tissues. a The increasing concentrations (0.125, 0.25 and 0.5 μg whole tissue extracts) of uracil incision (U:G) in NMIBC and their corresponding normal tissues (N). **b** Full length gels for uracil incision (U:G) activities in NMIBC and N tissues, and T24 BC cells. **c** UNG antibody inhibited uracil incision (U:G) activity. **d** Uracil incision (U:G) using purified UDG protein (1 U, lane 2). Uracil incision (U:G) in NMIBC (lane 5) and U:G incision in NMIBC tissue extract inhibited by ugi (lane 6). **e** Full-length Western blots. **f** The increasing concentrations of uracil incision activity in single-stranded oligonucleotides in T24 cells. **g** Full length gels for uracil incision activity in single-stranded oligonucleotides in NMIBC and N tissues.

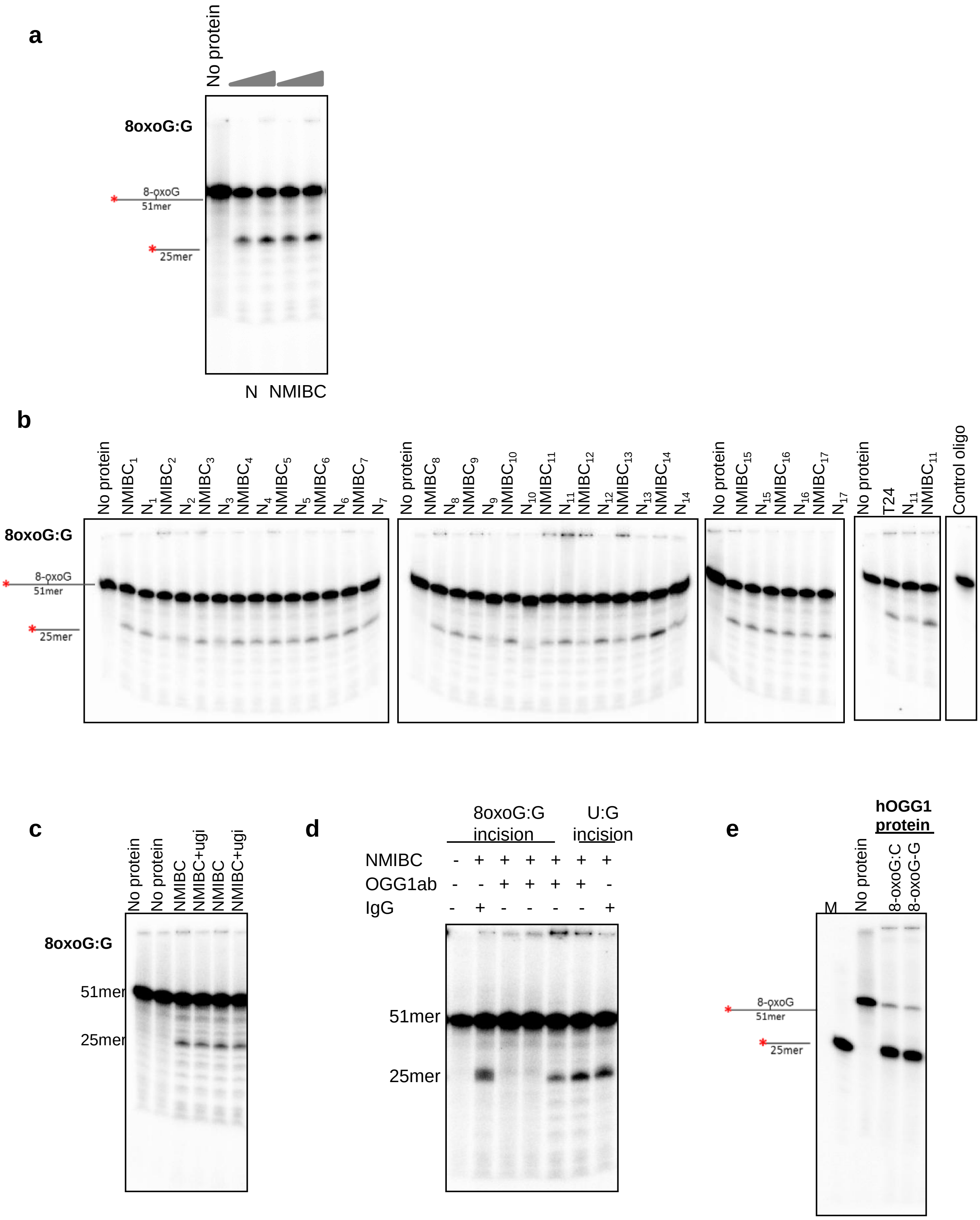

Supplementary Figure S3

f

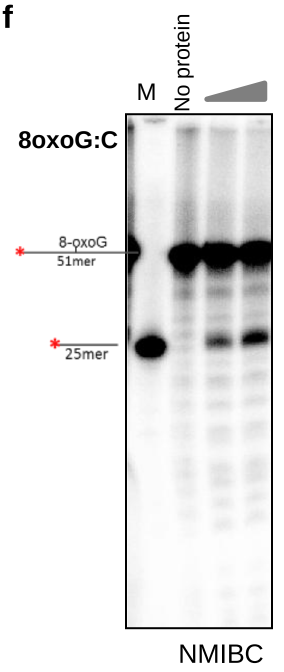

g

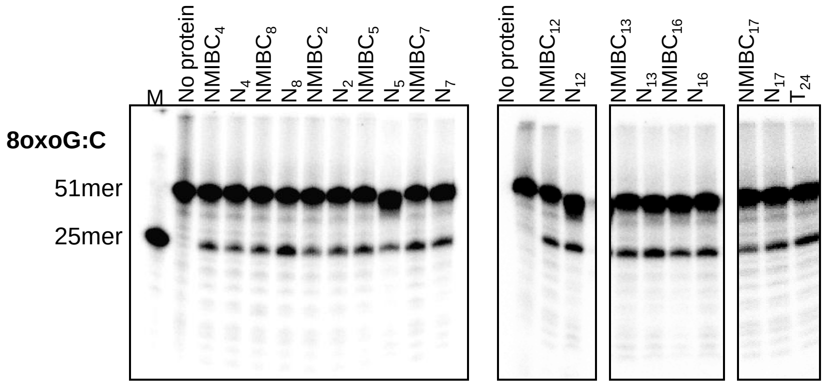

h

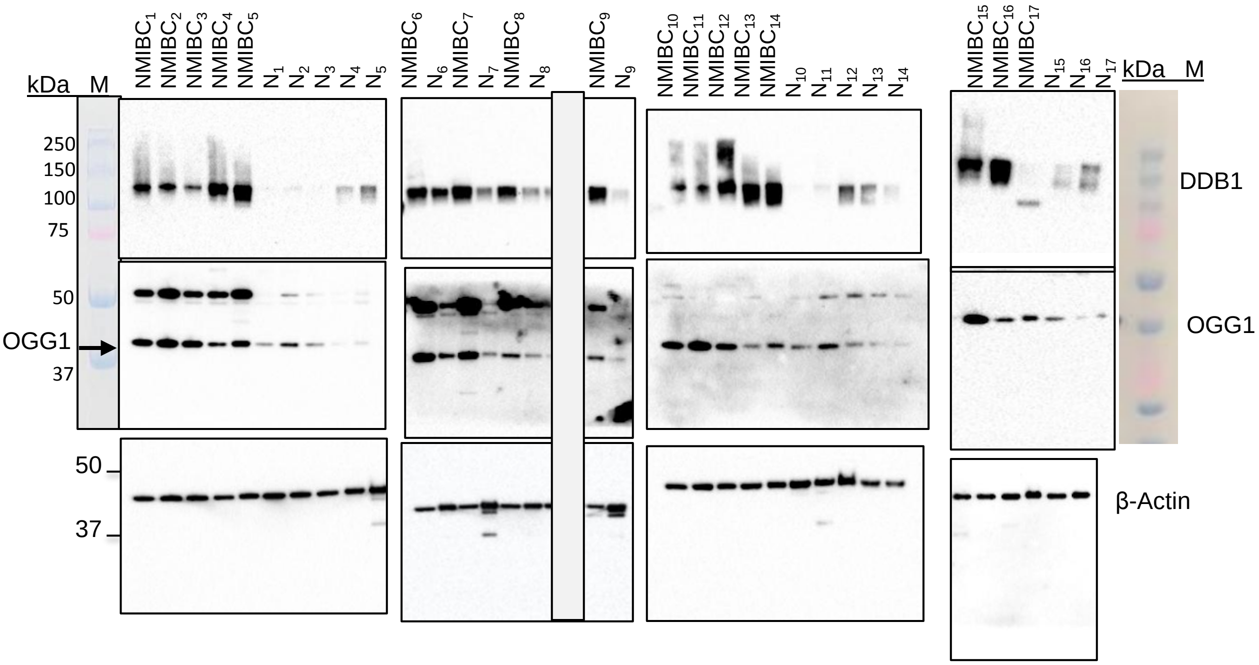

Supplementary Figure S3. 8-oxoG incision activity and OGG1 protein levels in NMIBC and their corresponding normal tissues. a The increasing concentration (0.5 and 1 μg whole tissue extracts) of 8-oxoG:G incision in NMIBC and the corresponding normal tissue (N). **b** Full length gels for 8-oxoG:G incision in NMIBC and N tissues, and T24 BC cells. **c** 8-oxoG:G incision in NMIBC tissue extracts with or without pre-incubation with ugi. **d** 8-oxoG:G and U:G incision activity in NMIBC tissue extracts with or without incubation with OGG1 antibody. **e** The incision of 8-oxoG:C and 8-oxoG-G substrates using purified hOGG1 protein (0.05 U). **f** The increasing concentration (0.5 and 1 μg whole tissue extracts) of 8-oxoG:C incision in NMIBC tissue extracts. **g** Full length gels for 8-oxoG:C incision in NMIBC and N tissues, and T24 BC cells. **h** Full length Western blots for OGG1 and DDB1.

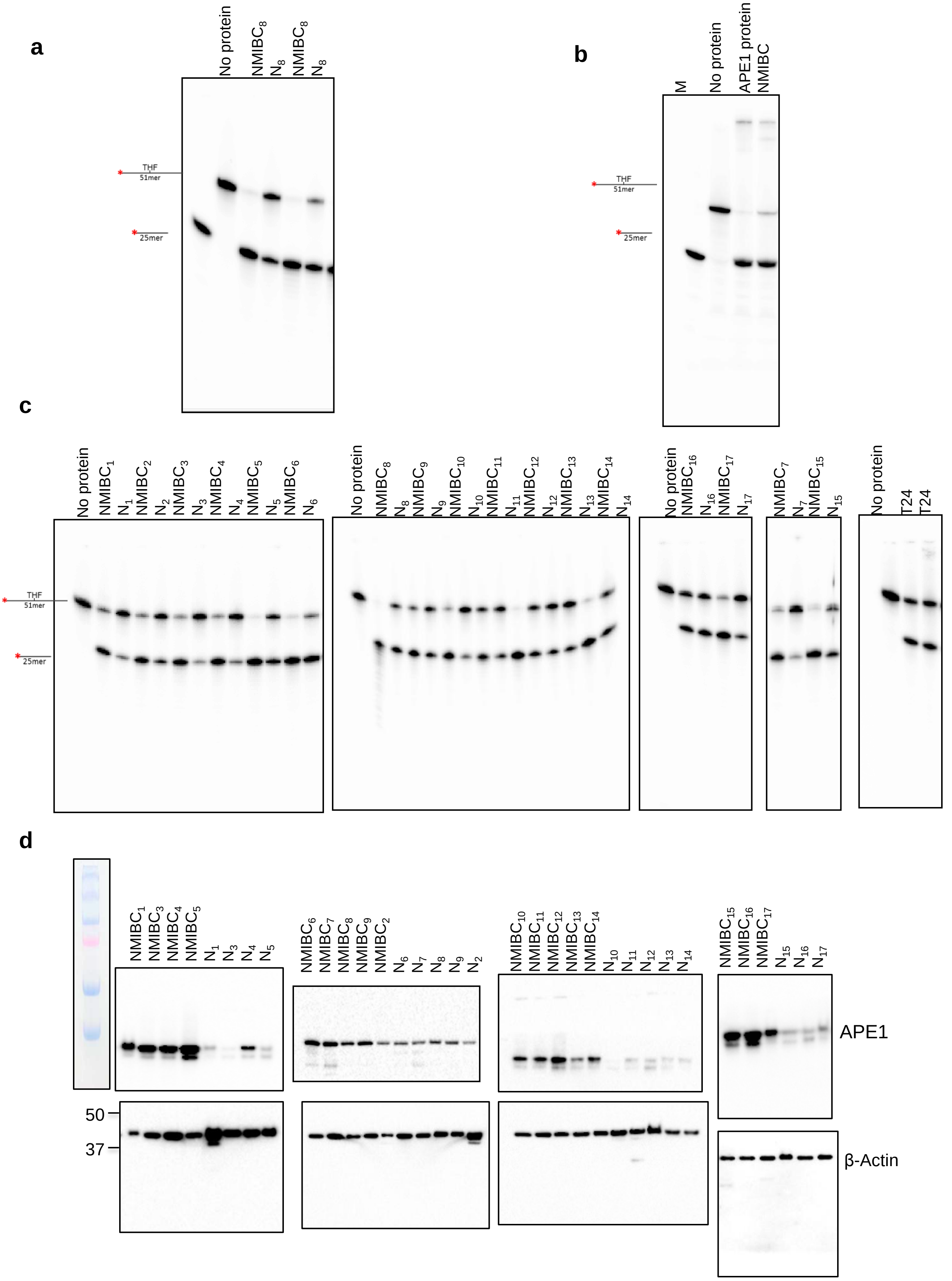

Supplementary Figure S4. APE1 activity and protein levels in NMIBC and the corresponding normal tissues. a The increasing concentration (0.02 and 0.05 μg whole tissue extracts) of THF incision in NMIBC and the corresponding normal tissues (N). **b** The incision of THF substrate using purified APE1 protein (0.05 U). **c** Full length gels for THF incision in NMIBC and N tissues, and T24 BC cells. **d** Full-length Western blots for APE1.

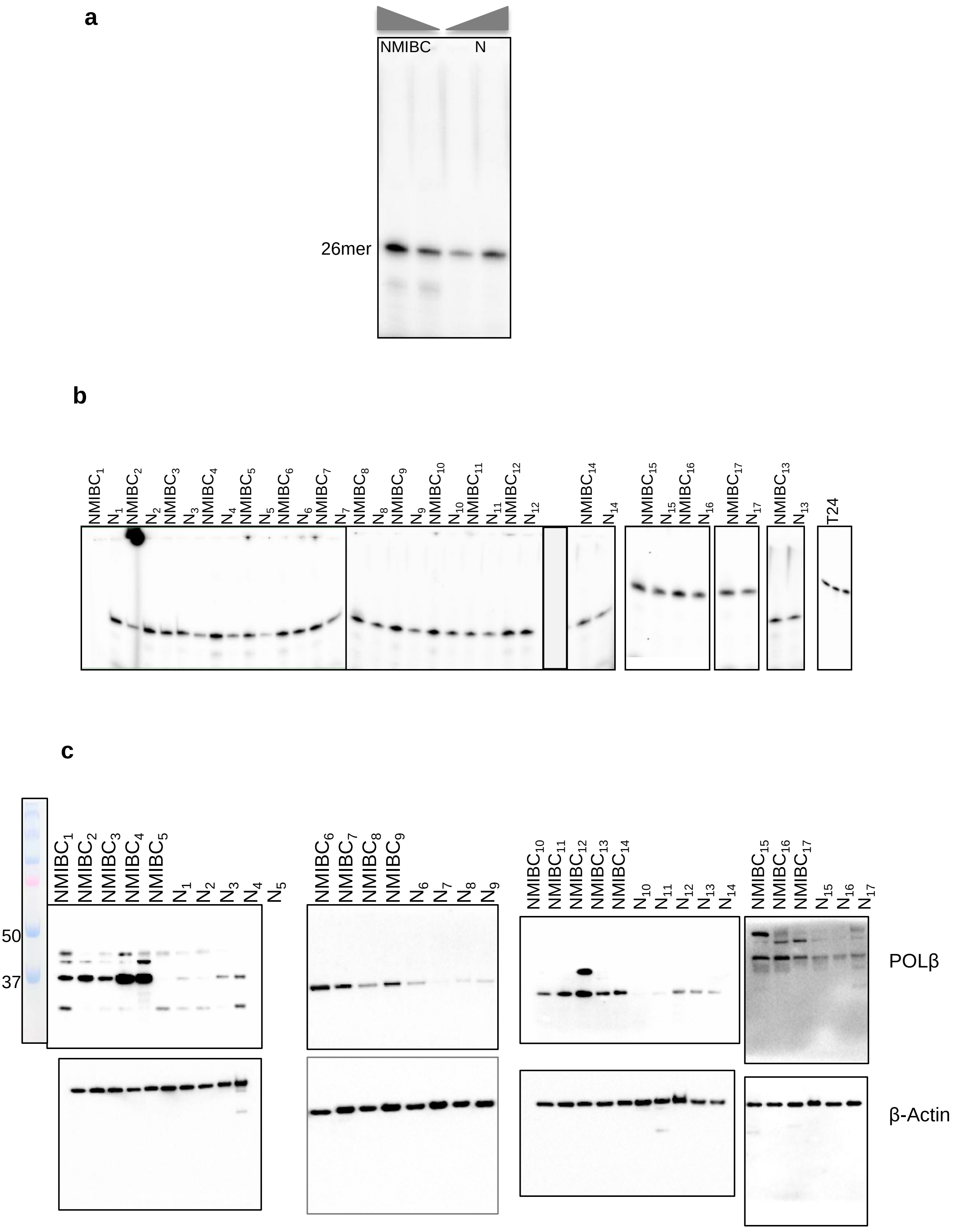

Supplementary Figure S5. a The increasing concentration (0.25 and 0.5 μg whole tissue extracts) of 1-nt gap filling in NMIBC and the corresponding normal tissues. **b** Full length gels for 1-nt gap filling in NMIBC and N tissues, and T24 BC cells. **c** Full length Western blots for POLβ.

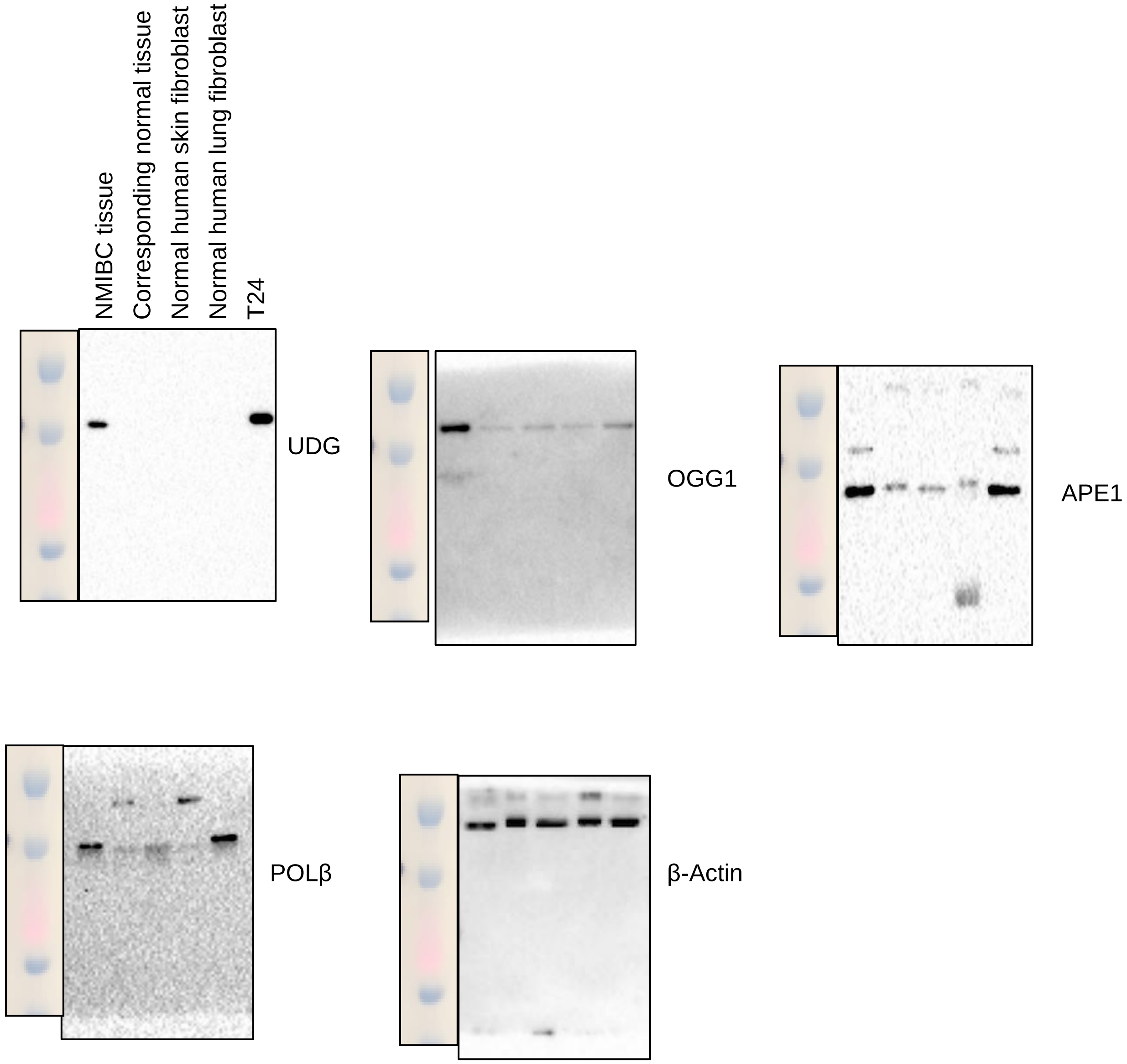

Supplementary Figure S6. BER protein levels in normal and NMIBC cells/tissues. Full length Western blots.

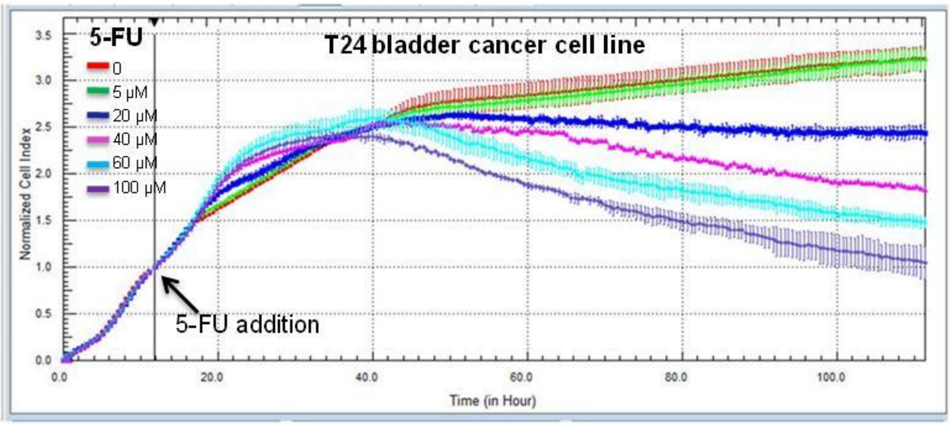

Time	T24, IC50 (μM)
24h	116
48h	290
72h	60.7
96h	42.3

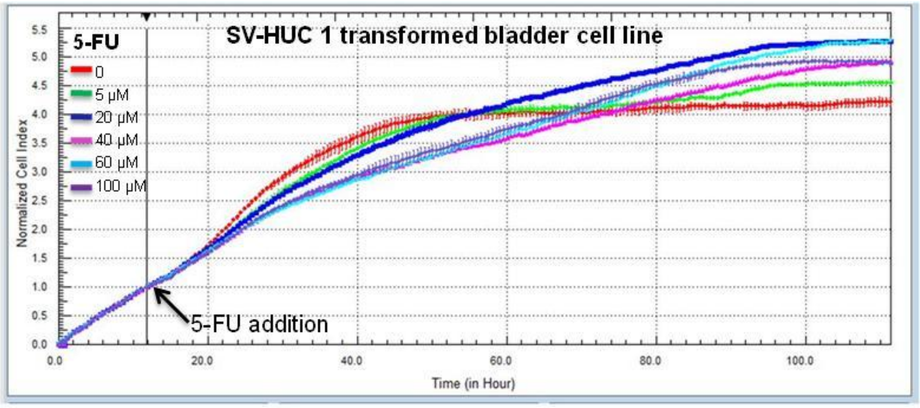

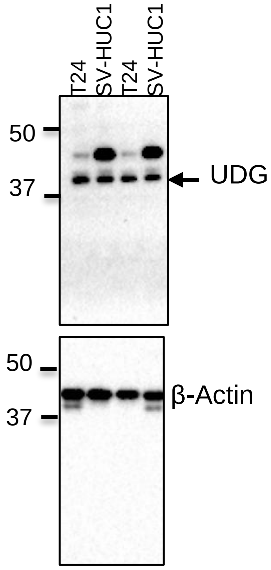

Supplementary Figure S7. Real-time dynamic monitoring of 5-fluorouracil (5-FU) cytotoxicity in T24 and SV-HUC-1 cell lines using the xCELLigence-DP system. To determine the effects of 5-FU on T24 and SV-HUC-1 cells (ATCC), 25,000 cells were seeded in an E-plate and when the cells were in the log growth phase, they were treated with various concentrations of 5-FU. Cell growth was continuously monitored every 30 min for 110 h. Cells treated with 0.01% DMSO were used as a control (red line). Cell index (CI) was normalized to the time point of 5-FU administration. Normalized CI was plotted as the mean value from triplicates; error bars represent the standard deviation of the mean. Black arrows indicate the time of 5-FU administration. The xCELLigence software used to determine IC50 values at four different post-treatment time points. Western blot analysis of UDG and β-actin in T24 and SV-HUC-1 cell lines.

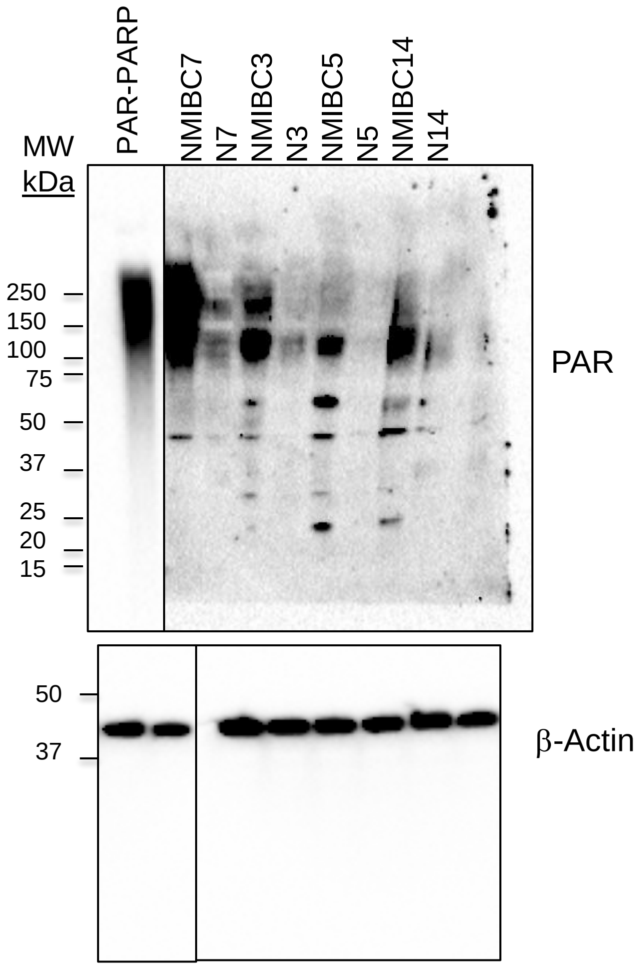

Supplementary Figure S8. Western blot analysis of parylated proteins in NMIBC and the corresponding normal tissue. Poly(ADP-ribose) (PAR) signal are detected between 15-250 kDa. PAR-PARP is parylated PARP protein was used as a control.
